# A systematic approach for authentication of medicinal *Patrinia* species using an integration of morphological, chemical and molecular methods

**DOI:** 10.1038/s41598-024-57115-w

**Published:** 2024-03-19

**Authors:** Kwan-Ho Wong, Tao Zheng, Grace Gar-Lee Yue, Man-Ching Li, Hoi-Yan Wu, Man-Ho Tong, Xin-Lei Zhao, Hu-Biao Chen, Clara Bik-San Lau, Pang-Chui Shaw, David Tai-Wai Lau

**Affiliations:** 1 Shiu-Ying Hu Herbarium, School of Life Sciences, The Chinese University of Hong Kong, Shatin, New Territories, Hong Kong SAR China; 2 School of Life Sciences, The Chinese University of Hong Kong, Shatin, New Territories, Hong Kong SAR China; 3 Li Dak Sum Yip Yio Chin R&D Centre for Chinese Medicine, The Chinese University of Hong Kong, Shatin, New Territories, Hong Kong SAR, China; 4 Institute of Chinese Medicine, The Chinese University of Hong Kong, Shatin, New Territories, Hong Kong SAR China; 5 State Key Laboratory of Research On Bioactivities and Clinical Applications of Medicinal Plants, The Chinese University of Hong Kong, Shatin, New Territories, Hong Kong SAR China; 6 The Institute of Medicinal Plant Development, The Chinese Academy of Medical Sciences and Peking Union Medical College, Haidian, Beijing, China; 7 School of Chinese Medicine, Hong Kong Baptist University, Kowloon Tong, Hong Kong SAR China; 8 Department of Pharmacology and Pharmacy & School of Chinese Medicine, Li Ka Shing Faculty of Medicine, The University of Hong Kong, Pokfulam, Hong Kong SAR China

**Keywords:** Biochemistry, Plant genetics

## Abstract

Four common *Patrinia* species, including *P. heterophylla*, *P. monandra*, *P. scabiosifolia* and *P. villosa*, have been documented as herbal medicines with various clinical applications, such as anti-cancer, anti-diarrhea and sedative. However, the authentication of medicinal *Patrinia* species poses a problem, particularly with the processed herbal materials. This study aimed to systematically authenticate the four medicinal *Patrinia* species in the market using morphological and chemical characterization, as well as DNA markers. We found the species identity authenticated by traditional morphologies were in good agreement with both chemical and molecular results. The four species showed species-specific patterns in chromatographic profiles with distinct chemical markers. We also revealed the power of complete chloroplast genomes in species authentication. The sequences of targeted loci, namely *atpB*, *petA*, *rpl2-rpl23* and *psaI-ycf4*, contained informative nucleotides for the species differentiation. Our results also facilitate authentication of medicinal *Patrinia* species using new DNA barcoding markers. To the best of our knowledge, this is the first report on the application of morphology, chemical fingerprinting, complete chloroplast genomes and species-specific Insertion-Deletions (InDels) in differentiating *Patrinia* species. This study reported on the power of a systematic, multidisciplinary approach in authenticating medicinal *Patrinia* species.

## Introduction

*Patrinia* species have been traditionally used by Chinese medicine practitioners for various kinds of disorders, especially colon cancer. In recent years, *Patrinia* species have been documented with a number of research in phytochemistry and pharmacology which are related to its traditional usage^1^. Specific anti-cancer studies were also documented from various research groups^2,3^. On the other hand, the herbs were usually adopted as food or supplements, hence they are widely cultivated in various provinces in China for various kinds of usage^4^. According to *Flora Reipublicae Popularis Sinicae* (FRPS), *Patrinia* has ten species, three subspecies and two varieties. In *Flora of China* (FOC), *Patrinia* has been classified as eleven species and three subspecies. Most of these species are believed to have medicinal value in general. However, only five *Patrinia* species, *P. scabiosifolia* Link, *P. scabra* Bunge, *P. heterophylla* Bunge, *P. villosa* (Thunb.) Juss. and *P. rupestris* (Pall.) Juss., were documented in *Zhonghua Bencao*^5^ and *Zhongyao Da Cidian*^6^, and their herbal materials were named as “Baijiang”, “Yanbaijiang” or “Mutouhui”. There is however no official record of its source plants and quality control requirement in the *Chinese Pharmacopoeia* (2020)^7^. Moreover, the market available *Patrinia* materials have never been well identified. Hence, there is a need to verify the source species of the market available *Patrinia* for their good quality control and clinical application.

When reviewing the preclinical research of *Patrinia* species, three species were usually adopted in most studies mainly due to their regional usage and retails availability. Firstly, *P. villosa* seems to be the most frequently used and researched in *Patrinia* history. The species was found to be used as traditional medicinal herbs, with various clinical applications in anti-cancer, anti-diarrhea, sedative, etc. Specific application was recorded for its treatment in colorectal cancer by activating the PI3K/Akt signaling pathway^8^. Secondly, *P. scabiosifolia* has quite abundant records of its usage relating to cancer. Various compounds or raw extracts of the herbs have been tested against human carcinoma cell lines, suggesting it to be a good anti-cancer herb^9,10^. Lignans, monoterpenes from the species also showed potential cytotoxic activities against human colon HCT-116 cells^11^. The herb was also found to inhibit the growth of 5-fluorouracil-resistant colorectal carcinoma cells^12^. Thirdly, *P. heterophylla* also has a number of records regarding its pre-clinical research. Its active components, including phenylpropanoids, flavonoid, iridoids and coumarins, also possessed cytotoxic activities against different tumor cells^13–15^.

It is important to note that the identity of the medicinal *Patrinia* species has always been confusing, and only few reports were found regarding their source materials authentication. One of the major concerns is the inconsistency of botanical description and the phenotypic structures. Variations of the authenticating characters, such as involucral bract and leaf segments, are usually noticed. More varying character states were found from the cultivated populations^16^. Moreover, medicinal *Patrinia* were applied as processed materials, including decoction pieces and concentrated granules. Hence, DNA fingerprints and chemical markers should be included as additional authentication tools to cope with various sample forms and increase the accuracy of authentication.

Chemical fingerprint is a reliable approach for TCM authentication as it supplements morphological evidence at another level of structural organization. Here, relations are investigated between different classes of plants and the occurrence of specific substances or substance groups in plant tissues^17,18^. Thin layer chromatography (TLC) has been regarded as an excellent tool for providing the chemical fingerprints. Compared to DNA fingerprinting and morphological identification, TLC is relatively simple, fast and inexpensive by fractionating complex plant extracts for their respective fingerprints, therefore for easy perceiving similarities among different plant species^19^.

Molecular authentication through DNA barcoding has been widely adopted for medicinal plants. Universal barcode regions, including nuclear ribosomal Internal Transcribed Spacer (nrITS), plastid *rbcL* and *psbA-trnH* intergenic spacer, are commonly used. However, their low differentiation power at species level^20^ and difficulties in amplification and sequencing^21^ have been reported. Meanwhile, the limited barcode sequences of *Patrinia* on NCBI GenBank were insufficient to allow meaningful comparisons and differentiation of these species. The study of Kim et al.^22^ attempted to differentiate four *Patrinia* species namely *P. scabiosifolia*, *P. villosa*, *P. saniculifolia* Hemsl. and *P. rupestris*, using three universal barcode regions namely ITS2, *matK* and *rbcL*. Their results showed that 22, 22, and 12 species-specific nucleotides in the amplicons of ITS2, *matK* and *rbcL*, respectively, could differentiate the four species. The study of Moon et al.^23^ developed molecular markers using random amplified polymorphic DNA (RAPD) genomic profiling, which the markers were designed for sequence characterized amplified region (SCAR). The above four species were successfully distinguished from each other through multiplex-PCR SCAR assays based on the molecular weight of amplicons. Yet, both studies did not perform phylogenetic analysis to confirm the monophyly of amplicon sequences and hence their species identity.

Authentication of traditional Chinese medicines (TCM) by chloroplast genomes was achieved by our research group, Yik et al.^24^ and Ngai et al.^25^ who authenticated Baihuasheshecao and Lingxiaohua, respectively. These studies confidently revealed the possibility of authenticating Baijiangcao (*Patrinia* species) by using complete chloroplast genomes.

The objective of this study is to systematically authenticate the commonly retailed *Patrinia* species using organoleptic structures, chemical fingerprints and DNA fingerprints. Four *Patrinia* species, namely *P. villosa* subsp. *villosa*. *P. scabiosifolia*, *P. heterophylla* and *P. monandra* C. B. Clarke, were found available in the market. Thirty-five samples were collected from various production sites in China, so the samples could well represent the current market status which truly reflect the TCM classes used by the consumers. It is believed that a comprehensive documentation of the marketable Patriniae Herba would be important for their quality control and standardization.

## Results

### Morphological authentication

**Figure 1:**
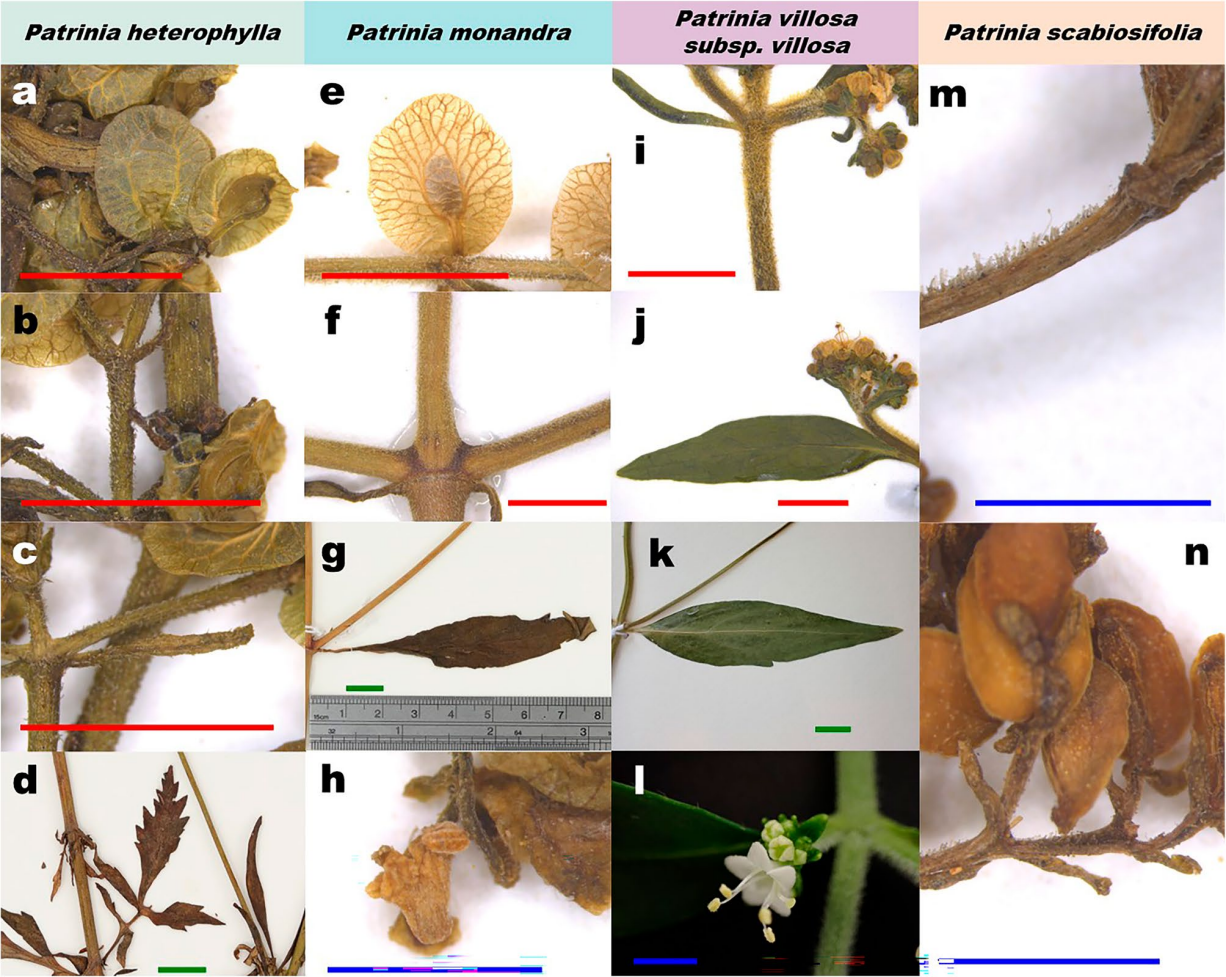
Key morphological characters for *Patrinia* species identification. (**a**–**d**) *P. heterophylla*, (**e**–**h**) *P. monandra*, (**i**–**l**) *P. villosa* subsp. *villosa*, (**m**,**n**) *P. scabiosifolia*. (**a**) Bracteole 2-veined, scale = 0.5 cm. (**b**) Peduncle densely hispidulous, scale = 0.5 cm. (**c**) Upper bract linear, scale = 0.5 cm. (**d**) Leaf with 2 pairs of segments, scale = 1.0 cm. (**e**) Bracteole 2-veined, scale = 0.5 cm. (**f**) Peduncle densely hirsute, scale = 0.5 cm. (**g**) Involucral bract around 8.5 cm, scale = 1.0 cm. (**h**) One longer stamen exserted, scale = 0.2 cm. (**i**) Peduncles densely hirsute, scale = 0.5 cm. (**j**) Involucral bract ovate-lanceolate, scale = 0.5 cm. (**k**) Cauline leaves no segment, scale = 1.0 cm. (**l**) Corolla white, scale = 0.2 cm. (**m**) Peduncles densely hirsute abaxially, scale = 0.2 cm. (**n**) Bracteole reduced, scale = 0.2 cm. Scar bars in blue = 0.02 cm, scale bars in red = 0.5 cm, scale bars in green = 1 cm.

The sample materials were well classified into 4 species, each has one representative specimen as reference voucher. The selected specimens were found to have critical characters that are consistent with the description of FRPS and FOC. To further confirm their identities, these plant structures were found to be matched with the authentication records given by other research groups^26,27^. The confirmed structural description with some key photos of the four species (Fig. 1) are given as below.

**(a)** ***Patrinia heterophylla*** **Bunge (M. C. Li 089)**

Bracteole 2-veined (Fig. 1a), ~ 6 mm; peduncle densely hispidulous (Fig. 1b); involucral bract in linear segment; upper bract linear (Fig. 1c); leave papery; basal leaves with two pairs of segments (Fig. 1d).

**(b)** ***Patrinia monandra*** **C. B. Clarke (M. C. Li 103)**

Bracteole 2-veined (Fig. 1e), ~ 5 mm; peduncle densely hirsute (Fig. 1f); involucral bract ~ 8.5 cm (Fig. 1g); one longer stamen exserted (Fig. 1h).

**(c)** ***Patrinia villosa*** **(Thunb.) Juss. subsp.** ***villosa*** **(M. C. Li 403)**

Bracteole 2-veined; peduncles densely hirsute (Fig. 1i); involucral bract ovate-lanceolate (Fig. 1j) to linear; basal leaves rosulate; cauline leaves no segment (Fig. 1k); corolla white (Fig. 1l).

**(d)** ***Patrinia scabiosifolia*** **Link (M. C. Li 083)**

Peduncles densely hirsute abaxially (Fig. 1m); bracteole reduced (Fig. 1n).

### Chemical analysis

To minimize unpredictable statistical bias in chemical fingerprint analysis, a total of 35 sample materials were performed. The R_*f*_ values were calculated using visionCATS software under UV light after spraying with a 10% sulfuric acid in ethanol. The use of UV 366 nm was found to be the most suitable for visualizing the compounds compared to ultraviolet radiation at 254 nm and white light. The chromatographic profiles indicated that all sample constituents were clearly separated without any tailing and diffuseness.

**Figure 2:**
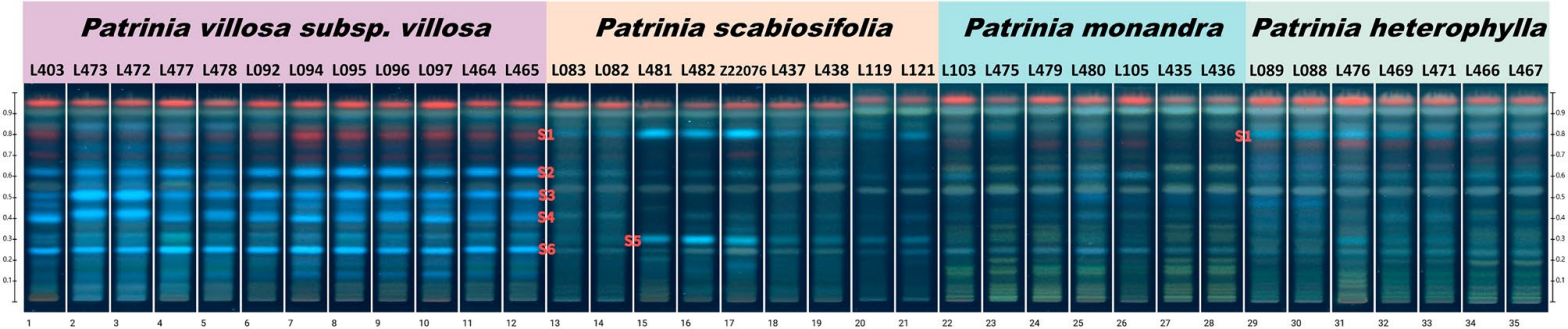
TLC Fingerprinting of *Patrinia* species under 366 nm UV light. TLC profiles of commodity samples of *P. villosa* subsp. *villosa* (tracks 1–12), *P. scabiosifolia* (tracks 13–21), *P. monandra* (tracks 22–28) and *P. heterophylla* (tracks 29–35) under UV at 366 nm after derivatization with 10% sulfuric acid in ethanol.

In general, the four different species were different in content and type of chemical components. The TLC fingerprints of *P. villosa* subsp. *villosa*, *P. scabiosifolia*, *P. heterophylla* and *P. monandra* are shown in Fig. 2. Distinct differences were observed among the chromatographic profiles at species level. The TLC fingerprint showed that there were four benchmark highlight blue spots S2, S3, S4 and S6, (R_*f*_ value 0.62, 0.51, 0.40 and 0.25, respectively) represented well the characteristics of *P. villosa* subsp. *villosa*. However, these four spots are absent in *P. scabiosifolia*. Further data analysis revealed that the spot S3 (R_*f*_ value 0.51) was the strongest spot in the TLC profile and only appeared in both *P. monandra* and *P. villosa* subsp. *villosa*, but the intensity was significantly lighter in *P. monandra*. These differences in the intensities of the spots representing the major compounds were evident within samples from different species. Similarly, the blue-green spot S1 (R_*f*_ value 0.81) was detected in *P. heterophylla* and *P. scabiosifolia*, but absent in *P. villosa* subsp. *villosa*. Therefore, the spot S1 should be unique to *P. scabiosifolia* and *P. heterophylla*. Interestingly, both the results of morphological identification and DNA fingerprinting indicated the species identity of sample M. C. Li 082 and M. C. Li 083 as *P. scabiosifolia*, but the TLC analysis revealed that these two samples lacked the spot S5 (R_*f*_ value 0.31), and the intensity of the spots S1 and S5 were also significantly weaker than other *P. scabiosifoilia* samples. Considering that the sample comes from different production sites, the potential reasons for this inter-individual variation could be caused by various factors such as genetic variability, environmental factors, and random chance. The results of TLC chromatograms for four different *Patrinia* species are summarized in Table 1. In conclusion, by examining the presence or absence of the spots S1–S6 in the TLC profile, as well as the intensity of the spot S3, we can eventually differentiate and identify these four different species.

**Table 1:** TLC pattern of methanol extracts of *Patrinia.*

Spot	S1	S2	S3	S4	S5	S6
RF	0.81	0.62	0.51	0.40	0.31	0.25
*P. villosa*	−	+	+	+	−	+
*P. scabiosifolia*	+	−	−	−	+ *	−
*P. monandra*	−	−	+	−	−	−
*P. heterophylla*	+	−	−	−	− ^#^	−

*Excluding M. C. Li 082 and M. C. Li 083, which the spot S5 was negative.

^#^Excluding M. C. Li 476, which the spot S5 was positive.

### Molecular analysis

#### Amplifiability of primers

Among the six pairs of designed primer, four pairs namely *Pat-rpl2-rpl23-2*, *Pat-petA*, *Pat-psaI-ycf4* and *Pat-atpB-1*, could successfully amplify targeted sequences from all four authenticated specimens. In contrast, the primer pairs *Pat-rpl2-rpl23-1* and *Pat-atpB-2* could amplify sequences from all authenticated specimens except the one of *Patrinia heterophylla* (M. C. Li 089). For fair comparison, we discarded these two primer pairs for the subsequent amplification and analysis of the thirty-three testing samples.

The two universal primer pairs, namely ITS-S2F/ITS-S3R and psbAF/trnHR, were able to amplify sequences from both the four authenticated specimens and the thirty-three testing samples. The differentiation power of both barcode regions universal for land plants and the targeted chloroplast regions for the genus *Patrinia* are discussed below.

#### Identification of species-specific SNPs and InDels

Species-specific variable nucleotides, including Single Nucleotides Polymorphisms (SNPs) and Insertion-Deletions (InDels), were identified from all studied taxon except *P. monandra* in the targeted chloroplast regions.

For *P. villosa* subsp. *villosa,* a species-specific insertion in 24 bp (3′ GAAGGGGTATGTTATTATTTTATT 5′) was found in the intergenic spacer of *psaI*-*ycf4* at the alignment position of 185th–208th bp (Table 2). Meanwhile, a species-specific substitution as cytosine (C) was found in the intergenic spacer of *rpl2-rpl23* at the 173rd alignment position, where other studied taxa shared the nucleotide guanine (G).

**Table 2:**
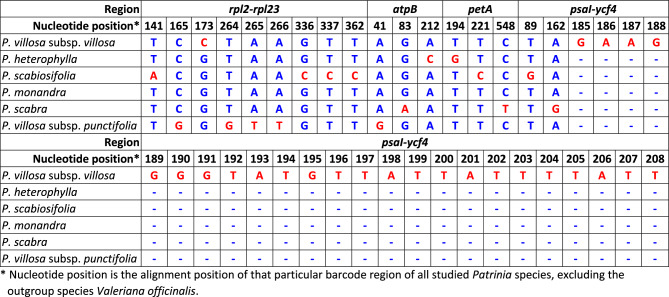
Species-specific variable nucleotides including Single Nucleotides Polymorphisms (SNPs) and Insertion-Deletions (InDels).

*Nucleotide position is the alignment position of that particular barcode region of all studied *Patrinia* species, excluding the outgroup species *Valeriana officinalis*.

For *P. scabiosifolia*, a total of six species-specific substitutions were found in the targeted chloroplast regions. One substitution as G was found in the intergenic spacer of *psaI-ycf4* at the 89th alignment position where other studied taxa shared the nucleotide thymine (T). Another substitution as adenine (A) was notified in the intergenic spacer of *rpl2-rpl23* at the 141st alignment position where other species shared the nucleotide T. In the same region, three other substitutions as C were found at the alignment positions of 336th, 337th and 362nd, where other species shared the nucleotides G, T and T, respectively. The last substitution as C was found in *petA* at the 221st alignment position, where other species shared the nucleotide T.

For *P. heterophylla*, two substitutions as C and G were respectively found in *atpB* at the alignment position of 212th and in *petA* at the alignment position of 194th, whereas other species shared the nucleotide A and T, respectively.

For *P. scabra*, three substitutions were found. In *atpB*, the substitution A was found in the alignment position of 83rd while other species shared the nucleotide G. At the alignment position 548th in *petA*, the substitution T of *P. scabra* was observed, in opposite to the nucleotide C in other species. In addition, the substitution G was found in *psaI-ycf4* at the alignment position of 162nd whereas other species shared the nucleotide A.

For *P. villosa* subsp. *punctifolia*, a total of five substitutions were found in the region *atpB* and *rpl2-rpl23*. One substitution G was found in the *atpB* at the alignment position of 41st, in opposite to the nucleotide A in other species. The other four substitutions, namely G, G, T and T, were respectively found in *rpl2-rpl23* at the alignment position of 165th, 264th, 265th and 266th, whereas other species shared the nucleotides C, T, A and A, accordingly.

Although there were no species-specific SNP or InDel observed in *P. monandra,* there is one “substitution-like” nucleotide (as A) at the very beginning of the *petA* alignment at the 24th position. Yet, at this early beginning of alignment, a few accessions of other *Patrinia* species showed no nucleotide, as a result we cannot regard this as a SNP.

The above SNPs and InDels, which differentiated the six taxa from each other, could be used as molecular diagnostic markers, particularly the long insertion of *P. villosa* subsp. *villosa* in *psaI*-*ycf4*.

#### Monophyly observed in single locus and multi-loci phylogenetic trees

Poor differentiation powers were seen from universal barcode regions of land plants namely ITS2 and *psbA-trnH*. In the Neighbour-Joining (NJ) tree constructed by ITS2, the accessions of different species clustered into multiple clades (Supplementary Fig. S1). The species resolution of *psbA-trnH* was low (as 33.3%; Table 3), yet the monophyly of *P. heterophylla* and *P. scabra* were confirmed in NJ tree (Supplementary Fig. S2). The combination of ITS2 and *psbA-trnH* were even worse than the single locus *psbA-trnH*, since only the cluster of *P. scabiosifolia* was monophyletic (Supplementary Fig. S3), suggesting these two universal regions are unable to authenticate *Patrinia* samples down to species-level.

**Table 3:** Information of each locus and loci combination for phylogenetic analysis.

Locus or combination of loci	Number of informative variable nucleotide (bp)	Number of species-specific variable nucleotide (bp)	Number of species having specific variable nucleotide(s)	Discrimination success rate (%)*	Best-fit model
ITS2	18	8	2	0.0	Tamura 3-parameter + Gamma distribution
*psbA-trnH*	22	9	2	33.3	Tamura 3-parameter + Invariable
ITS2 + *psbA-trnH*	40	17	3	16.7	Kimura 2-parameter + Gamma distribution
*atpB*	4	3	3	33.3	Kimura 2-parameter
*psaI-ycf4*	36	26	3	33.3	Tamura 3-parameter
*rpl2-rpl23*	20	9	3	50.0	Tamura 3-parameter + Gamma distribution
*petA*	12	3	3	100.0	Tamura 3-parameter
*psaI-ycf4* + *rpl2-rpl23*	56	35	4	33.3	Tamura 3-parameter + Gamma distribution
*atpB* + *psaI-ycf4*	40	29	5	66.7	Tamura 3-parameter
*petA* + *psaI-ycf4*	48	29	4	66.7	Tamura 3-parameter
*atpB* + *rpl2-rpl23*	24	12	5	83.3	Tamura 3-parameter + Gamma distribution
*atpB* + *petA*	16	6	4	100.0	Tamura 3-parameter
*petA* + *rpl2-rpl23*	32	12	5	100.0	Tamura 3-parameter + Gamma distribution
*atpB* + *psaI-ycf4* + *rpl2-rpl23*	60	38	5	83.3	Tamura 3-parameter + Gamma distribution
*atpB* + *petA* + *rpl2-rpl23*	36	15	5	100.0	Tamura 3-parameter + Gamma distribution
*petA* + *psaI-ycf4* + *rpl2-rpl23*	68	38	5	100.0	Tamura 3-parameter + Gamma distribution
*atpB* + *petA* + *psaI-ycf4*	52	32	5	100.0	Tamura 3-parameter
*atpB* + *petA* + *psaI-ycf4* + *rpl2-rpl23*	72	41	5	100.0	Tamura 3-parameter + Gamma distribution

* Discrimination success rate is equal to the number of monophyletic clade(s) formed in the NJ trees over the total number of studied taxa (as 6) times 100%.

Targeted chloroplast regions which were amplified using the designed primers showed improvement in differentiation power according to different loci combinations. The differentiation power of single locus region varies. The most powerful one was *petA* as all six taxa formed monophyletic clades in the NJ tree (Supplementary Fig. S4), with the greatest (100%) discrimination success rate (Table 3). It was noticed that *atpB* was able to differentiate *P. heterophylla* and *P. scabra* from the others (Supplementary Fig. S5), giving 33.3% discrimination rate. The region *psaI-ycf4* also showed 33.3% discrimination success rate, that *P. scabiosifolia* and *P. villosa subsp. punctifolia* were distinguished from the others. Interestingly, although a species-specific diagnostic marker of *P. villosa* subsp. *villosa* in 24 bp was found in this region, the subspecies was clustered with *P. monandra* in a large clade (Supplementary Fig. S6). Monophyletic clades of *P. scabiosifolia*, *P. villosa* subsp. *villosa* and *P. villosa* subsp. *punctifolia* were observed in the NJ tree of *rpl2-rpl23* (Supplementary Fig. S7), giving half (50%) discrimination success rate contributed by the twenty SNPs in this region, with nine of them being species-specific.

Among the six two-loci combinations, *atpB* + *petA* (Supplementary Fig. S8) and *petA* + *rpl2-rpl23* (Supplementary Fig. S9) showed the highest discrimination success rate (100%). Distinct monophyletic clades of the six studied taxa were observed in the NJ trees constructed by these two combinations. It was contributed by 16 and 32 informative variable nucleotides in the combination *atpB* + *petA* and *petA* + *rpl2-rpl23*, respectively, in which 6 and 12 of these nucleotides were species-specific. The other 4 two-loci combinations (Supplementary Figs. S10–S13) showed 33.3–83.3% discrimination success rate.

Among the four three-loci combinations, *petA* + *psaI-ycf4* + *rpl2-rpl23* (Supplementary Fig. S14), *atpB* + *petA* + *rpl2-rpl23* (Supplementary Fig. S15) and *atpB* + *petA* + *psaI-ycf4* (Supplementary Fig. S16) showed the highest discrimination success rate (100%). The combination *atpB* + *psaI-ycf4* + *rpl2-rpl23* (Supplementary Fig. S17) showed 83.3% discrimination success rate as the accessions of *P. monandra* and *P. villosa* subsp. *villosa* were clustered into one big clade in the NJ tree.

**Figure 3:**
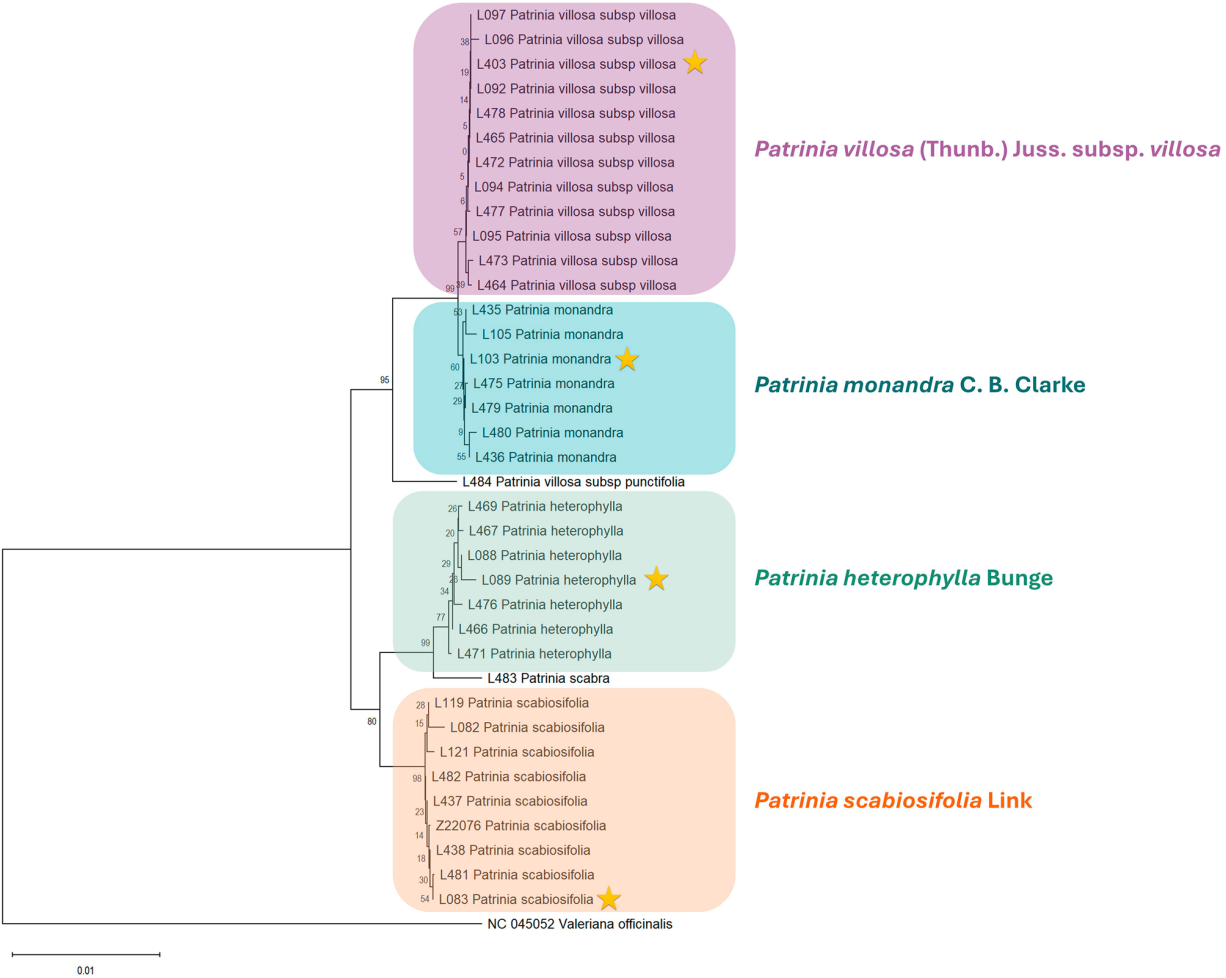
Neighbour-joining tree constructed from four-loci combination *atpB* + *petA* + *psaI-ycf4* + *rpl2-rpl23*. One unit in the scale bar 0.01 denoting the genetic distance. Asterisked accessions represented the authenticated specimens, while accessions not asterisked are the testing samples.

When all four regions were concatenated for NJ tree reconstruction, all six taxa were clearly divided into distinct monophyletic clades in the NJ tree (Fig. 3 and Supplementary Fig. S18). The topology of it was similar to those with 100% discrimination rate, namely *petA* + *rpl2-rpl23*, *atpB* + *petA* + *psaI-ycf4*, *atpB* + *psaI-ycf4* + *rpl2-rpl23*, *atpB* + *petA* + *rpl2-rpl23,* but not *petA and atpB* + *petA* which the cluster of *P. heterophylla* and *P. scabra* was not sister to *P. scabiosifolia*.

To explore the possible barcoding gaps, another genetic-distance based method Unweighted Pair Group Method with Arithmetic mean (UPGMA) was employed for phylogenetic reconstruction. The single locus *petA* showed six monophyletic clades for each taxa in the UPGMA tree (Supplementary Fig. S19), yet the topology was different from the NJ tree as the clade consisting of *P. heterophylla* and *P. scabra* became the basal one. The resolution of *atpB* had been increased in UPGMA tree (Supplementary Fig. S20) since *P. scabra*, *P. heterophylla* and *P. villosa* subsp. *punctifolia* were distinguished from the rest *Patrinia*. In the UPGMA tree of *rpl2-rpl23* (Supplementary Fig. S21), monophyletic clade of *P. monandra* was formed in contrary to the NJ tree, however *P. scabra* was still nested in the cluster of *P. heterophylla*. The resolution of *psaI-ycf4* was also improved in UPGMA tree (Supplementary Fig. S22) since all *Patrinia* taxa formed monophyletic clades, except *P. villosa subsp. villosa* and *P. monandra* were clustered into a large single clade. The UPGMA tree of four-loci combination (Supplementary Fig. S23) shared similar topology with the NJ tree that the six studied taxa were also in monophyly.

In addition to genetic-distance based method, character-based method as Maximum Likelihood (ML) was also employed. In the ML tree of *petA* (Supplementary Fig. S24), the clade of *P. monandra* was nested into the one of *P. villosa* subsp. *villosa*, while the rest taxa could be distinguished from each other. Paraphyly of four *Patrinia* taxa was observed in the ML tree of *atpB* (Supplementary Fig. S25), only *P. heterophylla* and *P. scabra* could form monophyletic clades, similar to the NJ tree. The resolution of the ML tree of *rpl2-rpl23* (Supplementary Fig. S26) was comparable to the NJ tree since the monophyly of *P. scabiosifolia*, *P. villosa* subsp. *villosa* and *P. villosa* subsp. *punctifolia* were observed, although the topology was slightly different as the cluster of *P. heterophylla* and *P. scabra* became the basal one. The ML tree of *psaI-ycf4* (Supplementary Fig. S27) was also similar to the NJ tree since only *P. scabiosifolia* and *P. villosa* subsp. *punctifolia* could be distinguished from the rest. The ML tree of four-loci combination (Supplementary Fig. S28) showed lower resolution than the NJ tree since *P. monandra* could not form monophyletic clade and clustered with *P. villosa* subsp. *villosa*.

## Discussion

Based on the 35 samples collected from different provinces in China, four species were identified including *P. villosa* (Thunb.) Juss. subsp. *villosa*, *P. scabiosifolia* Link, *P. heterophylla* Bunge and *P. monandra* C. B. Clarke. The first three species are also commonly recorded as medicinal species in *Patrinia* literatures. Moreover, the key morphological characters (Fig. 1) of the selected samples from the same species were consistent with the descriptions in the floras, showing stable phenotypic variations. The materials of these four species collected in this study were treated as reliable raw materials for further study in chemistry and DNA analysis. However, morphological variations of non-key characters were also observed among species and individuals, that required additional analyses other than morphological means for accurate authentication. Chemical method using thin layer chromatography (TLC) was adopted as the first consolidation of the species identify, since not all herbal forms show sufficient characters for morphological authentication.

The TLC technology, regarded as one of the fundamental and widely used chromatographic analysis methods, has been consistently applied in the chemical analysis of herbal medicines. The analytical approaches were well adopted by the Chinese Pharmacopeia owing to its simplicity, sensitivity and high throughput. With advancements in TLC equipment and automation, the fingerprinting method shows great potential for identifying and characterizing different species of plants. Our work has also successfully revealed the distinct differences among the chemical fingerprints of *P. villosa* subsp. *villosa*, *P. scabiosifolia*, *P. monandra*, and *P. heterophylla*. These characteristic TLC profiles of plant species not only aid in their identification and quality control, but also provide information for further identification of chemical marker compounds specific to each species. Therefore, based on the different TLC fingerprints of *Patrinia* (as shown in Table 1), future research will focus on isolating and identifying chemical markers such as S1, S3, and S5, which can be used to differentiate and identify the four species of *Patrinia.*

Repeatedly analysis of the TLC profiles showed the five spots with different R_*f*_ values were mostly consistent in most samples of the same species, which could be adopted as authenticating chemical fingerprints. Compared with DNA authentication, time of developing TLC profile was comparatively shorter, which is about two-third less for the same quantity of samples. However, the composition of phytochemistry is prone to be affected by various factors, including growing stages, medicinal parts used, growth environment and post-harvest processing. These factors contributed to the observed inconsistency in the spot intensity as in our results (Fig. 2), that two *P. scabiosifolia* (M. C. Li 082 and 083) samples showed comparatively weaker intensity of spot S1 and S5, while one *P. heterophylla* (M. C. Li 476) showed greater intensity of spot S5 which were even absent in other samples of *P. heterophylla*. Moreover, if two herbs are closely related, their chemical profiles of great similarity could not be differentiated.

In order to develop a platform of accurate authentication, DNA analysis was further employed. The time cost of DNA analysis was greatly increased by a list of sequential procedures, i.e. DNA extraction, PCR amplification, purification of PCR products, Sanger sequencing and nucleotide analyses including SNP and InDel analysis and phylogenetic tree reconstruction. The completion of all procedures could take about two to three weeks. Yet, species identity could be reinforced critically by the species-specific SNPs and InDels, as well as the monophyly of singe-species clades in phylogenetic tree. More importantly, this molecular evidence is less influenced by environmental factors when comparing to chemical markers. The integration of the three authentication methods by morphological, chemical and molecular evidence contributed to an accurate authentication platform of *Patrinia* herbal medicines.

In this study, the application of complete chloroplast genomes in authenticating plant species was fully demonstrated. The method and results are valuable in DNA barcoding authentication, particularly for the plant taxa that are hardly differentiated down to species-level by using universal barcode regions. As in the case of *Patrinia* species, the universal barcode regions were not useful in differentiating the targeted four species through DNA barcoding. In this study, although eighteen variable nucleotides including eight species-specific nucleotides were identified in the universal barcode region ITS2, it showed no resolution in species discrimination. It is contrasting to the work of Kim et al.^22^ that 22 species-specific nucleotides were found in this nuclear region. The reason could be probably due to different primer pairs were used and different species were studied. The primer pairs ITS-S2F and ITS-S3R were used in this study, while ITS-S2F and ITS4 were adopted in the study of Kim et al.^22^. Besides, *P. heterophylla*, *P. monandra*, *P. scabra* and *P. villosa* subsp. *punctifolia* were included in this study, but not *P. saniculifolia* and *P. rupestris* in the study of Kim et al.^22^.

When using the targeted regions based on the full alignment of the complete chloroplast genomes, the sequence of amplicons could truly help in species differentiation, although the discrimination success rate of each targeted regions varies. It is suggested to use *petA* as the key barcode region as this single region has genetic information to differentiate all six studied taxa into monophyletic clades. When combining two loci for phylogenetic reconstruction, both combination of *atpB* + *petA* and *petA* + *rpl2-rpl23* could increase the discrimination success rate up to 100%, further revealing the importance of *petA* in species authentication. When three loci were used for phylogenetic reconstruction, the combination *atpB* + *petA* + *rpl2-rpl23, petA* + *psaI-ycf4* + *rpl2-rpl23 and atpB* + *petA* + *psaI-ycf4* provided the best discrimination success rate (100%). So, if limited resources are obtained, it is suggested to simply use the single locus *petA* for differentiating *Patrinia* species. However, 2 to 3 loci should be considered in order to provide better resolution. Particularly, the species-specific insertion in 24 bp (3′ GAAGGGGTATGTTATTATTTTATT 5′) of *P. villosa* subsp. *villosa* is highly informative. Although having the same discrimination success rate as 33.3% with *psaI-ycf4*, the region *atpB* is not preferred as only 4 informative variable nucleotides were found. The region *rpl2-rpl23* has twenty informative variable nucleotides in which nine of them are species-specific, contributing to the moderate discrimination success rate as 50%. Therefore, *psaI-ycf4* and *rpl2-rpl23* should be considered as auxiliary markers for better resolution and greater bootstrap values.

The topological differences between NJ, UPGMA and ML trees of the four-loci combination were probably caused by the lack of species-specific nucleotides in distinguishing *P. monandra* from the others. It was obvious that monophyletic clade of *P. monandra* could not be formed in the ML tree. Future study in capturing species-specific chloroplast SNPs and InDel of *P. monandra* and other *Patrinia* species would be helpful in increasing the resolution of phylogenetic analyses.

Comparing to the RAPD genomic profiling and SCAR markers in the study of Moon et al.^23^, the utilization of complete chloroplast genomes in authenticating medicinal *Patrinia* is relatively reliable and stable. Firstly, heavy work in screening suitable markers and primers were required in Moon’s study. Forty-seven out of eighty-six Operon primers produced distinct RAPD profiles, with twenty-eight primers showing polymorphic fragments. Based on forty-six species-specific amplicons, forty-three SCAR primer pairs were designed and eight of them were selected to capture the species-specific amplicons. In contrast, primer design and selection using chloroplast genome is more convenient and less labor intensive. Demonstrated in this study, three divergence hotspots were identified on chloroplast genomes alignment, and 4 out of 6 primer pairs were screened through amplification trial. Secondly, the multiplex-SCAR assay was restricted by the spectrum of amplicon size, as the primer sets had been chosen to visualize the differentiation in species-specific size. In our study, the amplicons were simply purified from agarose gel for sequencing, phylogenetic tree reconstruction and identification of informative nucleotides. Thirdly, the stability and specificity of RAPD profiling is affected by PCR conditions. In contrast, all PCR amplifications for targeted chloroplast regions were conducted under the same condition, with relatively low annealing temperature as 40 °C that is less specific, yielded strong bands at desired amplicon size for most of the samples as shown in our electrophoresis gels (Supplementary Fig. S29–S36). In addition, chloroplast genomes allow us to design specific primers capturing DNA fragments originated from chloroplast, and hence avoiding fungal contamination which occurs in ITS.

In summary, this study truly reflects the power of integrating plant taxonomy, chemical fingerprinting and DNA analysis. It is hard to start any authentication without knowing the name of the plants and the DNA sequences. Traditional morphological identification of plant species is the most efficient and direct way of authentication, but phytochemicals would become important markers when morphology or genomic DNA are not available. When samples quality is good enough for DNA extraction, the power of complete chloroplast genomes was demonstrated in breaking through the limitation of universal barcode regions. This study is also the first time to discover long fragment of species-specific InDels in the chloroplast regions for species differentiation of *Patrinia*. In conclusion, the three aspects of authentication methods would complement to each other to cope with various samples forms and states for better quality control of Chinese medicines.

## Methods

### Morphological authentication

Fresh samples of *Patrinia* species available in the markets were purchased from various provinces in China (Table 4). All fresh parts with flowers or fruits were used to prepare herbarium specimens. The identity of each sample was morphologically confirmed by studying the characters of the bracteole, peduncle indumentum, involucral bract, leave texture, basal or cauline leave arrangement, stamen structure and corolla color.

**Table 4:** Herbal materials used for morphological, chemical and molecular authentication.

Collector number	Code	Authenticated species	Province/municipality^#^	City/district/autonomous perfecture*	County	Category
M. C. Li 083	L083	*Patrinia scabiosifolia* Link	Hunan	Yongzhou	Jianghua Yao Autonomous County	Authenticated specimen
M. C. Li 089	L089	*Patrinia heterophylla* Bunge	Shanxi	Shangluo	Danfeng County	
M. C. Li 103	L103	*Patrinia monandra* C. B. Clarke	Hunan	Xiangxi Tujia & Miao Autonomous Prefecture	Fenghuang County	
M. C. Li 403	L403	*Patrinia villosa* (Thunb.) Juss. subsp. *villosa*	Zhejiang	Wenzhou	Rui'an County-level City	
M. C. Li 082	L082	*Patrinia scabiosifolia* Link	Hunan	Yongzhou	Jianghua Yao Autonomous County	Testing samples
M. C. Li 088	L088	*Patrinia heterophylla* Bunge	Shanxi	Shangluo	Danfeng County	
M. C. Li 092	L092	*Patrinia villosa* (Thunb.) Juss. subsp. *villosa*	Chongqing^#^	Liangping District	Zhushan Town	
M. C. Li 094	L094	*Patrinia villosa* (Thunb.) Juss. subsp. *villosa*	Zhejiang	Lishui	Jinyun County	
M. C. Li 095	L095	*Patrinia villosa* (Thunb.) Juss. subsp. *villosa*	Fujian	Sanming	Taining County	
M. C. Li 096	L096	*Patrinia villosa* (Thunb.) Juss. subsp. *villosa*	Jiangxi	Ji'an	Suichuan County	
M. C. Li 097	L097	*Patrinia villosa* (Thunb.) Juss. subsp. *villosa*	Hunan	Yongzhou	Qiyang County-level City	
M. C. Li 105	L105	*Patrinia monandra* C. B. Clarke	Hunan	Huaihua	Mayang Miao Autonomous County	
M. C. Li 119	L119	*Patrinia scabiosifolia* Link	Hunan	Yongzhou	Jianghua Yao Autonomous County	
M. C. Li 121	L121	*Patrinia scabiosifolia* Link	Guizhou	Qianxinan Buyei and Miao Autonomous Prefecture	Anlong County	
M. C. Li 435	L435	*Patrinia monandra* C. B. Clarke	Hunan	Xiangxi Tujia & Miao Autonomous Prefecture	Fenghuang County	
M. C. Li 436	L436	*Patrinia monandra* C. B. Clarke	Hunan	Xiangxi Tujia & Miao Autonomous Prefecture	Fenghuang County	
M. C. Li 437	L437	*Patrinia scabiosifolia* Link	Hunan	Yongzhou	Jianghua Yao Autonomous County	
M. C. Li 438	L438	*Patrinia scabiosifolia* Link	Hunan	Yongzhou	Jianghua Yao Autonomous County	
M. C. Li 464	L464	*Patrinia villosa* (Thunb.) Juss. subsp. *villosa*	Hubei	Huanggang	Yingshan County	
M. C. Li 465	L465	*Patrinia villosa* (Thunb.) Juss. subsp. *villosa*	Hubei	Huanggang	Yingshan County	
M. C. Li 466	L466	*Patrinia heterophylla* Bunge	Shanxi	Shangluo	Danfeng County	
M. C. Li 467	L467	*Patrinia heterophylla* Bunge	Shanxi	Shangluo	Danfeng County	
M. C. Li 469	L469	*Patrinia heterophylla* Bunge	Shanxi	Shangluo	Danfeng County	
M. C. Li 471	L471	*Patrinia heterophylla* Bunge	Shanxi	Shangluo	Danfeng County	
M. C. Li 472	L472	*Patrinia villosa* (Thunb.) Juss. subsp. *villosa*	Hubei	Huanggang	Yingshan County	
M. C. Li 473	L473	*Patrinia villosa* (Thunb.) Juss. subsp. *villosa*	Hubei	Huanggang	Yingshan County	
M. C. Li 475	L475	*Patrinia monandra* C. B. Clarke	Hunan	Xiangxi Tujia & Miao Autonomous Prefecture	Fenghuang County	
M. C. Li 476	L476	*Patrinia heterophylla* Bunge	Henan	Zhengzhou	Gongyi County-level City	
M. C. Li 477	L477	*Patrinia villosa* (Thunb.) Juss. subsp. *villosa*	Fujian	Longyan	Liancheng County	
M. C. Li 478	L478	*Patrinia villosa* (Thunb.) Juss. subsp. *villosa*	Hunan	Yongzhou	Jianghua Yao Autonomous County	
M. C. Li 479	L479	*Patrinia monandra* C. B. Clarke	Sichuan	Leshan	Jinkouhe District	
M. C. Li 480	L480	*Patrinia monandra* C. B. Clarke	Guizhou	Qiandongnan Miao & Dong Autonomous Prefecture	Jianhe County	
M. C. Li 481	L481	*Patrinia scabiosifolia* Link	Liaoning	Benxi	Huanren Manchu Autonomous County	
M. C. Li 482	L482	*Patrinia scabiosifolia* Link	Guizhou	Guiyang	Huaxi District	
M. C. Li 483	L483	*Patrinia scabra* Bunge	Henan	Gongyi	Xigui Highway	
M. C. Li 484	L484	*Patrinia villosa* (Thunb.) Juss. subsp. *punctifolia* H. J. Wang	Liaoning	Benxi	Huanren Manchu Autonomous County	
X. L. Zhao 22076	Z22076	*Patrinia scabiosifolia* Link	Hubei	Huanggang	Hong'an County	

*Unless specified, the listed regions are city-level.

^#^Chongqing is one of the Direct-administered municipalities in China.

All authenticated samples after standardized specimen processing methods were deposited in the Shiu-Ying Hu Herbarium (herbarium code: CUHK) as voucher specimens with collector numbers. Materials for chemical and molecular analysis were reserved for each specimen. The samples were well classified into 6 taxa. Among all samples, four specimens with well preserved and clear structures were adopted as our reference specimens (authenticated specimens in Table 4). The collector numbers are given as below:

*Patrinia heterophylla* Bunge (M. C. Li 089)

*Patrinia monandra* C. B. Clarke (M. C. Li 103)

*Patrinia scabiosifolia* Link (M. C. Li 083)

*Patrinia villosa* (Thunb.) Juss. subsp. *villosa* (M. C. Li 403)

### Chemical authentication

For each of the herbal samples, the test solution was prepared by extracting 2 g dried and pulverized herb with 20 ml methanol under ultrasonic condition at room temperature (approximately 21 °C) for 60 min, followed by filtration. The filtrate was then evaporated to dryness under reduced pressure at 50 °C. The extract was dissolved in 5 ml of methanol and was used for TLC analysis on silica gel 60 F_254_ TLC plates (20 cm × 10 cm, Merck, Germany). Extracts (2 μL) were applied to the plates as 8 mm bands using the CAMAG automatic TLC Sampler 4 (ATS4, Muttenz, Switzerland), development to a distance of 8.5 cm up the plate was performed in a TLC developing chamber. A mixture of ethyl acetate: methanol: water (8:1:1, v/v, upper layer) was used as the developing solvent system. The plate was then heated on a TLC plate heater (CAMAG, Muttenz, Switzerland) at about 105 °C after spraying with the 10 % solution of sulfuric acid in ethanol until the color of the spots appeared distinctly. High-definition images of the TLC plate were captured using a Visualizer 3 (CAMAG, Muttenz, Switzerland) linked with WinCATS software^28^ under UV light (λ = 366 nm).

### Molecular authentication

#### Sliding window analysis and primer design

**Figure 4:**
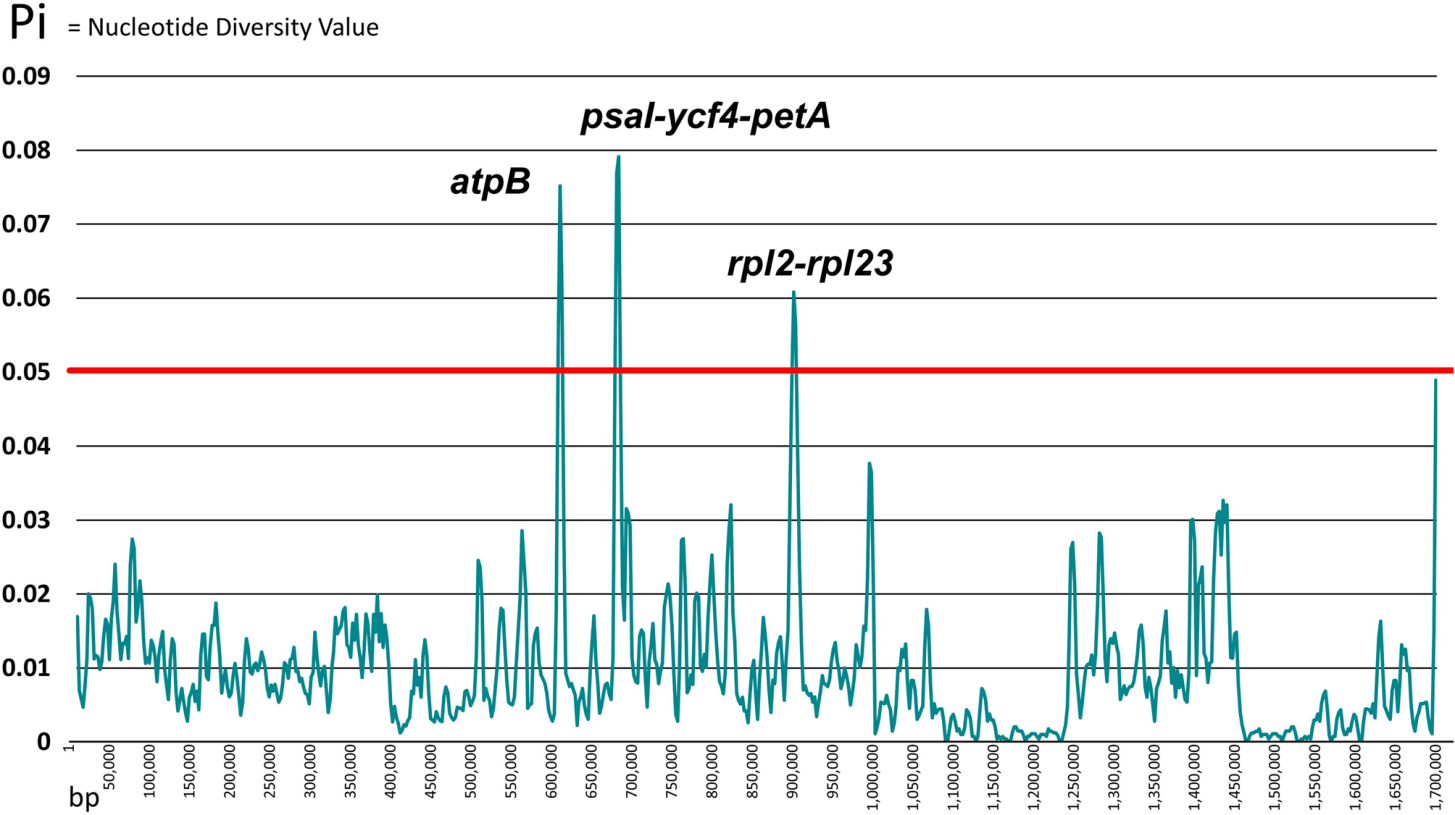
Identification of hotspot regions through sliding window analysis. The X-axis represents the alignment positions of the complete chloroplast genomes. The Y-axis marks the value of nucleotide diversity values (Pi). In total three hotspot regions—*atpB*, *psaI-ycf4-petA* and *rpl2-rpl23*—were identified with the threshold Pi > 0.05 as the red line.

The nine accessions of *Patrinia* complete chloroplast genomes (Supplementary Table S1) available on NCBI GenBank were downloaded for alignment. MAFFT version 7^29^ were used to align the chloroplast genomes. Sliding window analysis was performed using DNA Sequence Polymorphism (DnaSP) version 6.12.03^30^ for the calculation of nucleotide diversity values (Pi) from the aligned chloroplast genomes, in which the window length and the step size were set to 600 bp and 200 bp, respectively. The result was then visualized in a line chart (Fig. 4). Hotspot regions were identified with a threshold value Pi = 0.05. The loci above this value were considered as potential candidates for species differentiation.

Three hotspots were identified, namely *atpB* (alignment position: 57,125–58,621 bp), *psaI-ycf4*-*petA* (66,232–69,774 bp) and *rpl2-rpl23* (94,020–95,295 bp). All the hotspot regions were located in Large Single Copy (LSC). Since the hotspot *psaI-petA* in over 3500 bp was too long for PCR amplification, only the hypervariable regions were targeted, resulted in two loci as *psaI*-*ycf4* (66,232–67,096 bp) and *petA* (69,520–69,774 bp) were considered for primer design.

According to the hotspot regions, six pairs of primers were designed (Table 5) to capture the hypervariable positions with differentiating power. Since the intergenic spacer between the protein-coding gene *rpl2* and *rpl23* exceed 1200 bp which was not beneficial for PCR amplification, two pair of primers were designed to amplify two separated fragments (630 bp and 460 bp) of this locus. The same treatment was also performed for the loci *atpB* since this protein-coding gene exceeds 1400 bp.

**Table 5:** Primer pairs used for PCR amplification.

Pair	Name	Direction	Primer name	Sequence	Melting temperature (°C)	Target amplicon size (bp)
1	*ITS-S2F/ITS-S3R*	Forward	ITS-S2F	ATGCGATACTTGGTGTGAAT	55.13	500
		Reverse	ITS-S3R	GACGCTTCTCCAGACTACAAT	57.49	
2	*psbAF/trnHR*	Forward	psbAF	GTTATGCATGAACGTAATGCTC	56.23	300
		Reverse	trnHR	CGCGCATGGTGGATTCACAATCC	64.97	
3	*Pat-rpl2-rpl23-1*	Forward	Pat-rpl2-rpl23-F1	TCCAAGTGCAGGATAACC	53.79	630
		Reverse	Pat-rpl2-rpl23-R1	GATGTCTCCTACGTTACC	51.13	
4	*Pat-rpl2-rpl23-2*	Forward	Pat-rpl2-rpl23-F2	GGTCGTATTCTATGGTTACG	53.10	460
		Reverse	Pat-rpl2-rpl23-R2	ACTTCTAATGTCGAATCAGG	52.44	
5	*Pat-petA*	Forward	Pat-petA-FP	GGAACAGATTACTCGATCC	52.26	735
		Reverse	Pat-petA-RP	ACATTGGGATTACTCGTC	51.23	
6	*Pat-psaI-ycf4*	Forward	Pat-psaI-ycf4-FP	GGTGTAACATATGGCTTCC	53.10	340
		Reverse	Pat-psaI-ycf4-RP	TACTTGATCCGGTTGCAT	53.46	
7	*Pat-atpB-1*	Forward	Pat-atpB-F1	GGTTGTGATAAGAAACGC	51.51	430
		Reverse	Pat-atpB-R1	GCAGGATCTGAAGTATCTG	52.22	
8	*Pat-atpB-2*	Forward	Pat-atpB-F2	CAGATCCTGCTTGGACGA	56.34	420
		Reverse	Pat-atpB-R2	CCTATTCATAGATCTGCGCC	55.58	

#### DNA extraction

About 50 mg of each silicon-dried leaf sample (Table 4) were taken for DNA extraction (Supplementary Table S2). Weighed samples were placed in 2 mL Precellys Hard tissue grinding MK28 (Bertin Corp., Maryland, USA), and homogenized by Precellys Evolution Tissue Homogenizer (Bertin Technologies, Montigny-le-Bretonneux, France) using hard tissue mode. Total genomic DNA of all studied samples were extracted using i-genomic Plant DNA Extraction Mini Kit (iNtRON Biotechnology, Daejeon, Korea) following the instructions of the manufacturer. The quality and quantity of extracted DNA were assessed by 1.5 % agarose gel electrophoresis and NanoDrop Lite Spectrophotometer (Thermo Fisher Scientific, Massachusetts, USA), respectively.

#### PCR amplification, agarose gel electrophoresis and DNA sequencing

PCR amplification using both designed and universal primer pairs (Table 5) was firstly conducted for the samples of the four authenticated specimens representing *P. scabiosifolia* (M. C. Li 083), *P. villosa* subsp. *villosa* (M. C. Li 403), *P. monandra* (M. C. Li 103) and *P. heterophylla* (M. C. Li 089) (Table 4). After assessing the amplifiability, the primer pairs were used to amplify the target sequences from thirty-three testing samples collected from various locations in the mainland China. These samples include six samples of *P. heterophylla*, six samples of *P. monandra*, eight samples of *P. scabiosifolia* and eleven samples of *P. villosa* subsp. *villosa*. In addition, to test the amplifiability on other *Patrinia* taxa, a sample of *P. scabra* Bunge and one of *P. villosa* subsp. *punctifolia* H. J. Wang were adopted for PCR amplification using the selected primer pairs.

Extracted total genomic DNA of each sample were amplified using GoTaq^®^ G2 Flexi DNA Polymerase (Promega, Wisconsin, USA). In each 30-μL reaction, 6 μl 1X Green GoTaq^®^ Flexi Buffer, 3 μl MgCl2 (2.5 mM), 0.6 μl Promega dNTPs mix (0.2 mM), 1.5 μl Forward Primer (500 nM), 1.5 μl Reverse Primer (500 nM), 0.2 μl GoTaq polymerase (1 U/μl), 1 μL template DNA and 16.2 μl double-distilled water were included. Thermocycling procedures were undertaken in Applied Biosystems VeritiPro 96-Well Thermal Cycler (Thermo Fisher Scientific, Massachusetts, USA), started with an incubation at 95 °C for 4 minutes, followed by 35 cycles of denaturation at 95 °C for 30 s, annealing at 40 °C (or 45°C for ITS2 and *psbA-trnH*) for 30 s and elongation at 72 °C for 40 s, and finished by a final extension at 72 °C for 4 min. PCR products were kept at 12 °C or stored at 4 °C refrigerator until being subjected to gel electrophoresis in 1.5 % agarose gels for purification. QIAquick Gel Extraction Kit (Qiagen Co., Hilden, Germany) were used to purify PCR products following manufacturer’s instructions. Purified PCR products were sent to Tech Dragon Limited (Shatin, Hong Kong, China) for Sanger sequencing using Applied Biosystems 3730xl DNA Analyzer. Bidirectional sequences were assembled using CodonCode Aligner (Centerville, Massachusetts, USA)^31^. All assembled sequences were uploaded to NCBI GenBank, with the accession number of OR712158 to OR712225, PP277662 to PP277698 and PP280905 to PP281021 (Supplementary Table S3). Low-quality nucleotides with QV value below 30 at the two ends were discarded.

#### Phylogenetic analysis

Single locus and multiple-loci combination of sequences were used for phylogenetic analysis to assess their differentiation power down to species level. The sequences were firstly aligned using MAFFT version 7^29^, and then being adopted for phylogenetic tree construction using MEGA X version 10.2.5^32^. The best-fit model with the lowest Bayesian Information Criterion (BIC) was selected. To explore the possible barcoding gaps of the four targeted chloroplast loci, genetic-distance based methods namely Neighbour-Joining (NJ) and Unweighted Pair Group Method with Arithmetic mean (UPGMA), as well as character-based method i.e. Maximum Likelihood (ML), were adopted for phylogenetic analysis of the studied *Patrinia* species. For the multiple-loci combination, the amplicon sequences of each specimen were accordingly concatenated into a single sequence, which were then aligned using MAFFT version 7^29^. NJ, UPGMA and ML trees were constructed from single locus and four-loci combinations, while only NJ trees were constructed from two-loci and three-loci combinations.

To root the trees, *Valeriana officinalis* L. from the family Caprifoliaceae was selected as an outgroup species. Fragments of ITS2 and all chloroplast regions (*psbA-trnH*, *atpB*, *petA*, *rpl2-rpl23* and *psaI-ycf4*) were extracted from the NCBI accessions ON685480 (3713 bp) and NC_045052 (complete chloroplast genome in 151,505 bp^33^), respectively. To further prove the authentication power by this method, the two additional well-authenticated *Patrinia* taxa, *P. scabra* Bunge (M. C. Li 483) and *P. villosa* (Thunb.) Juss. subsp. *punctifolia* H. J. Wang (M. C. Li 484), were included in the phylogenetic analysis.

Informative variable nucleotides, including species-specific and non-species-specific nucleotides, were manually identified using BioEdit^34^ based on the unrooted alignments of each locus (Table 3). These variable nucleotides were classified as Single-Nucleotide Polymorphisms (SNPs) and Insertions–deletions (Indels). The number of variable nucleotides of multi-loci combinations were then calculated. Discrimination success rates was calculated by dividing the number of monophyletic clades containing single taxon in the NJ tree over the total number of studied *Patrinia* taxon (as 6) times 100%.

### Supplementary Information

Supplementary Information.

## Data Availability

All DNA barcode sequences amplified from the studied *Patrinia* species with voucher specimens were submitted to and available in the GenBank Database (https://www.ncbi.nlm.nih.gov/nuccore). Accession numbers: OR712158–OR712225, PP277662–PP277698 and PP280905–PP281021. The accession number corresponds to the six specified regions for each specimen were listed in Supplementary Table S3.
