# Supplementary material for: A systematic approach for authentication of medicinal Patrinia species using an integration of morphological, chemical and molecular methods
Source: Sci Rep. 2024 Mar 19;14:6566. doi: 10.1038/s41598-024-57115-w (PMC10951358; doi:10.1038/s41598-024-57115-w)
Supplement: Supplementary file 1 — Supplementary Information. [file 41598_2024_57115_MOESM1_ESM.pdf]

A systematic approach for authentication of medicinal *Patrinia* species using an integration of morphological, chemical and molecular methods

Supplementary materials

Kwan-Ho Wong[1,2,3,#], Tao Zheng[4,5,#], Grace Gar-Lee Yue[4,5], Man-Ching Li[1], Hoi-Yan Wu[3], Man-Ho Tong[4,5], Xin-Lei Zhao[6], Hu-Biao Chen[7], Clara Bik-San Lau [3,4,5,8,*], Pang-Chui Shaw[2,3,4,5,**], David Tai-Wai Lau [1,2,3,***]

[1] Shiu-Ying Hu Herbarium, School of Life Sciences, The Chinese University of Hong Kong, Shatin, New Territories, Hong Kong SAR, China;

[2] School of Life Sciences, The Chinese University of Hong Kong, Shatin, New Territories, Hong Kong SAR, China;

[3] Li Dak Sum Yip Yio Chin R & D Centre for Chinese Medicine, The Chinese University of Hong Kong, Shatin, New Territories, Hong Kong SAR, China;

[4] Institute of Chinese Medicine, The Chinese University of Hong Kong, Shatin, New Territories, Hong Kong SAR, China;

[5] State Key Laboratory of Research on Bioactivities and Clinical Applications of Medicinal Plants, The Chinese University of Hong Kong, Shatin, New Territories, Hong Kong SAR, China;

[6] The Institute of Medicinal Plant Development, the Chinese Academy of Medical Sciences and Peking Union Medical College, Haidian, Beijing, China;

[7] School of Chinese Medicine, Hong Kong Baptist University, Kowloon Tong, Hong Kong SAR, China;

[8] Department of Pharmacology and Pharmacy & School of Chinese Medicine, Li Ka Shing Faculty of Medicine, The University of Hong Kong, Pokfulam, Hong Kong SAR, China.

[#] The authors contributed equally.

Corresponding authors:
* Clara Bik-San Lau, Institute of Chinese Medicine & State Key Laboratory of Research on Bioactivities and Clinical Applications of Medicinal Plants, The Chinese University of Hong Kong, Shatin, New Territories, Hong Kong SAR, China.

Email: claralau@cuhk.edu.hk

** Pang-Chui Shaw, School of Life Sciences, Li Dak Sum Yip Yio Chin R & D Centre for Chinese Medicine, Institute of Chinese Medicine & State Key Laboratory of Research on Bioactivities and Clinical Applications of Medicinal Plants, The Chinese University of Hong Kong, Shatin, New Territories, Hong Kong SAR, China.

Email: pcshaw@cuhk.edu.hk

*** David Tai-Wai Lau, Shiu-Ying Hu Herbarium, School of Life Sciences, The Chinese University of Hong Kong, Shatin, New Territories, Hong Kong SAR, China.

Email: lautaiwai@cuhk.edu.hk

Fig. S1 – Neighbour-joining tree constructed from single DNA barcode region ITS2

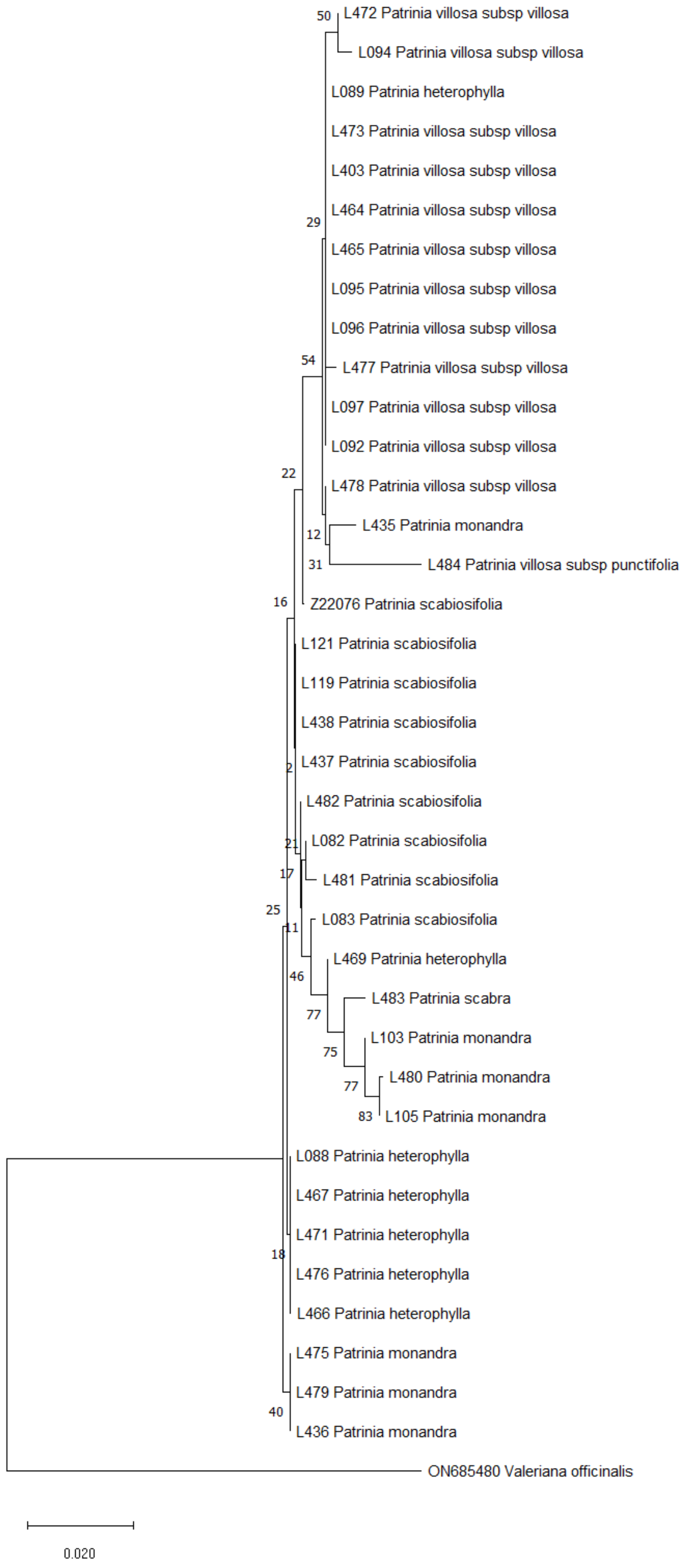

Fig. S2 – Neighbour-joining tree constructed from single DNA barcode region *psbA-trnH*

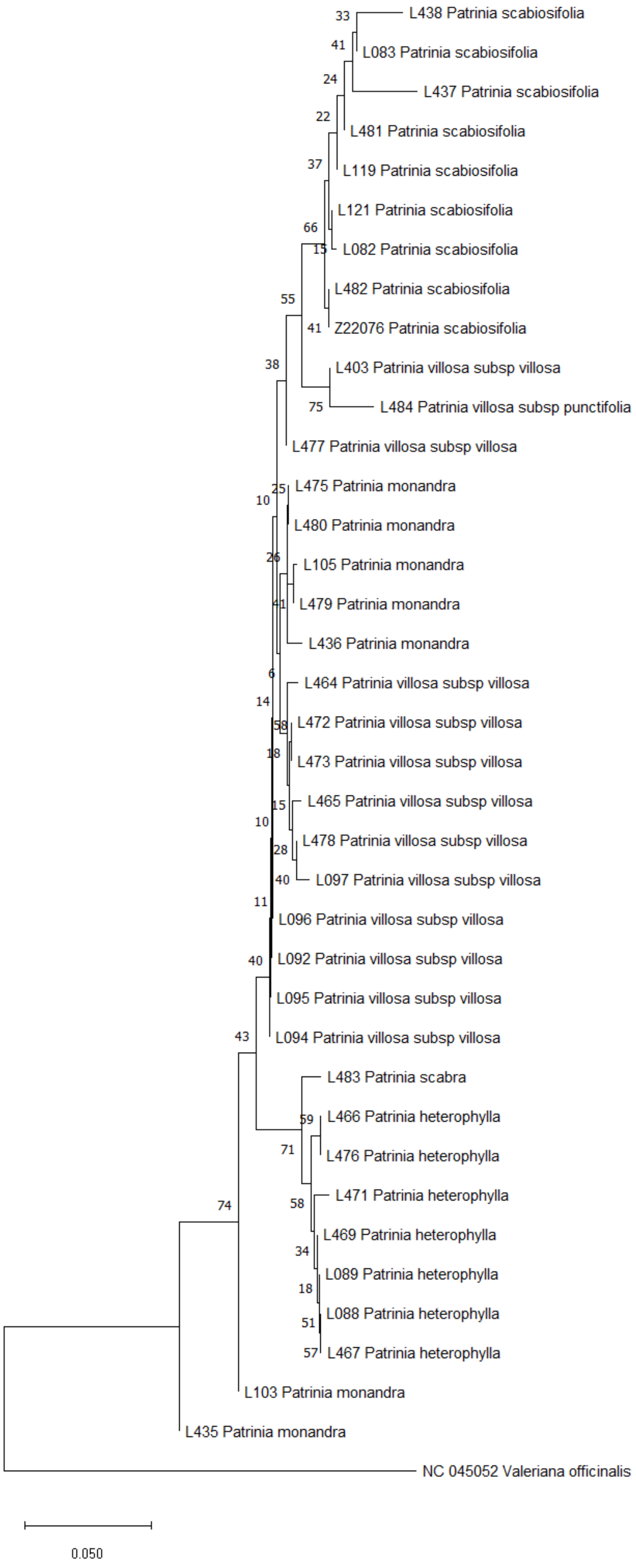

Fig. S3 – Neighbour-joining tree constructed from two-loci combination ITS2+*psbA-trnH*

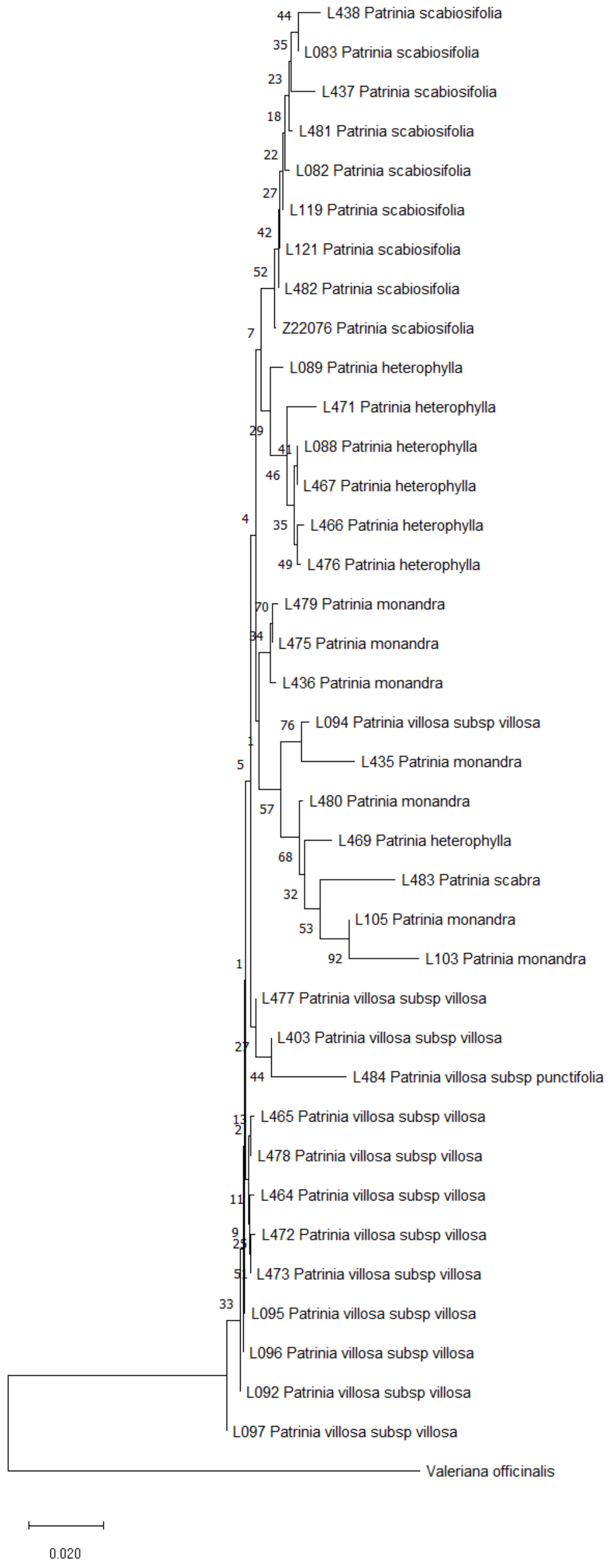

Fig. S4 – Neighbour-joining tree constructed from single DNA barcode region *petA*

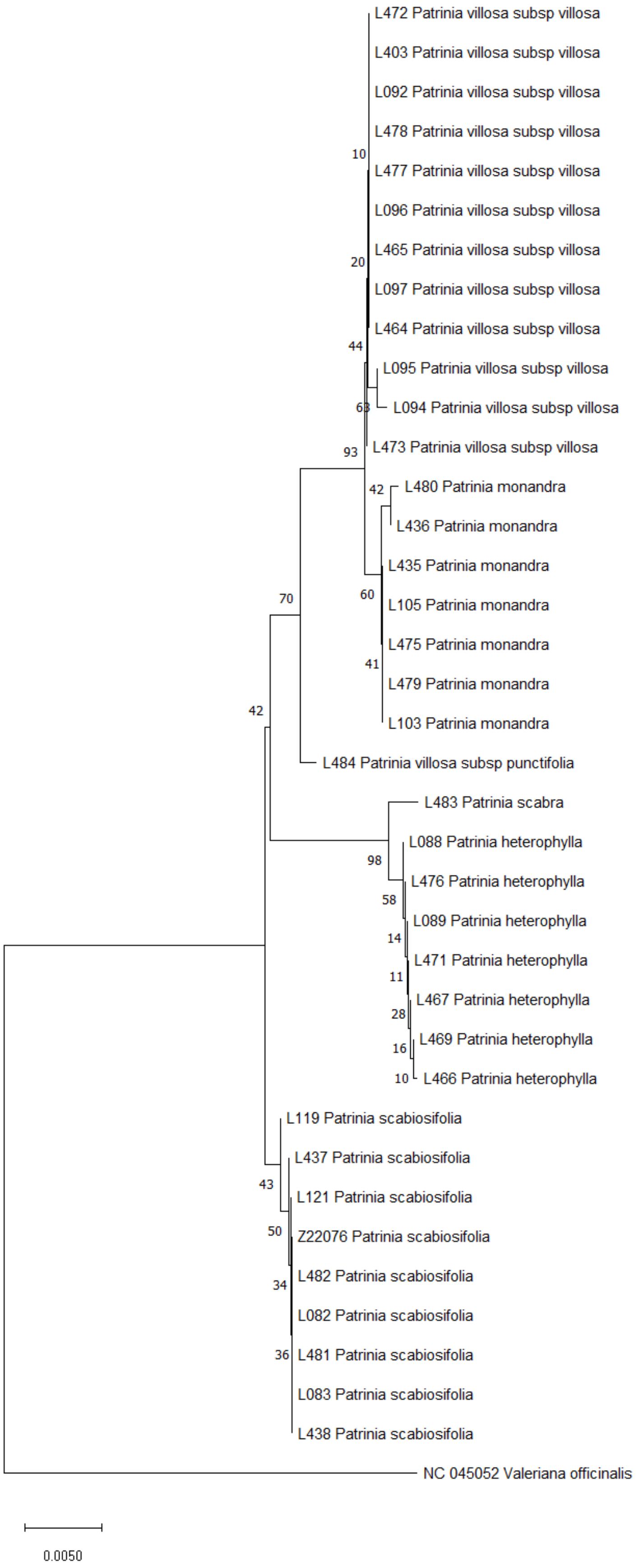

Fig. S5 – Neighbour-joining tree constructed from single DNA barcode region *atpB*

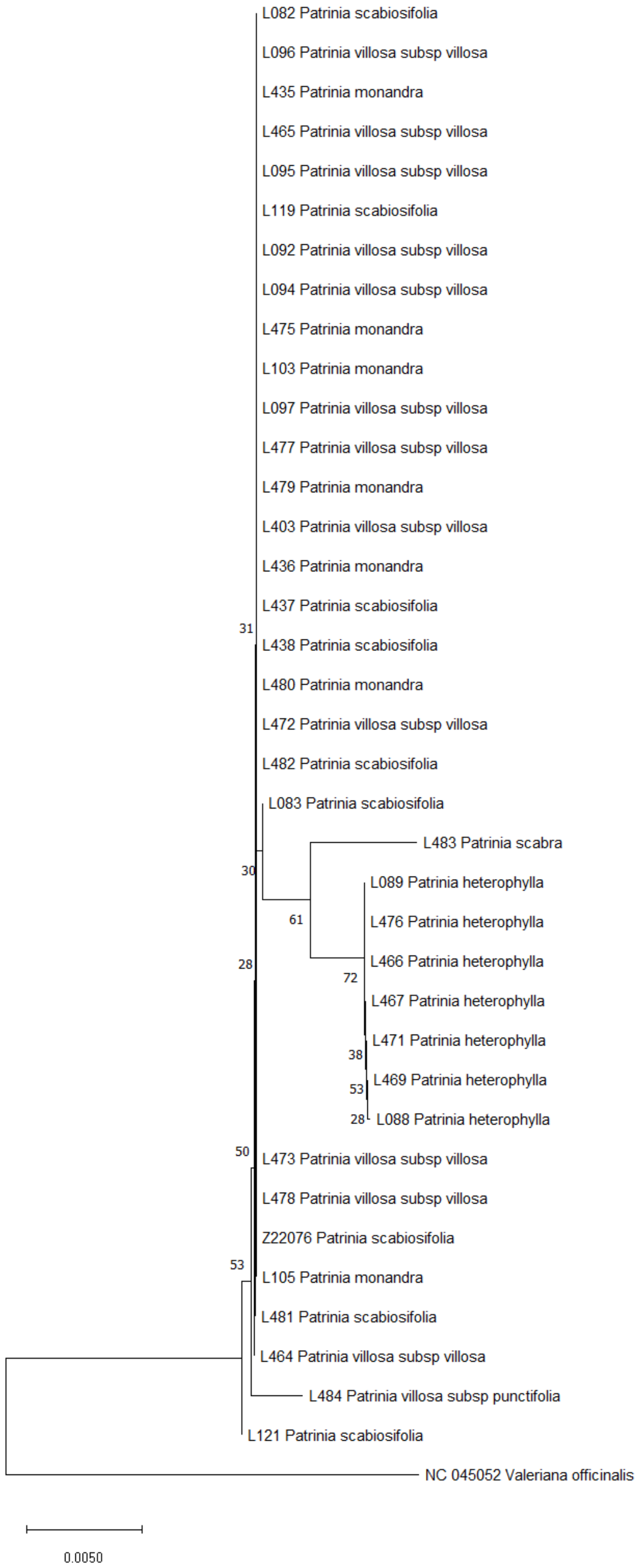

Fig. S6 – Neighbour-joining tree constructed from single DNA barcode region *psaI-ycf4*

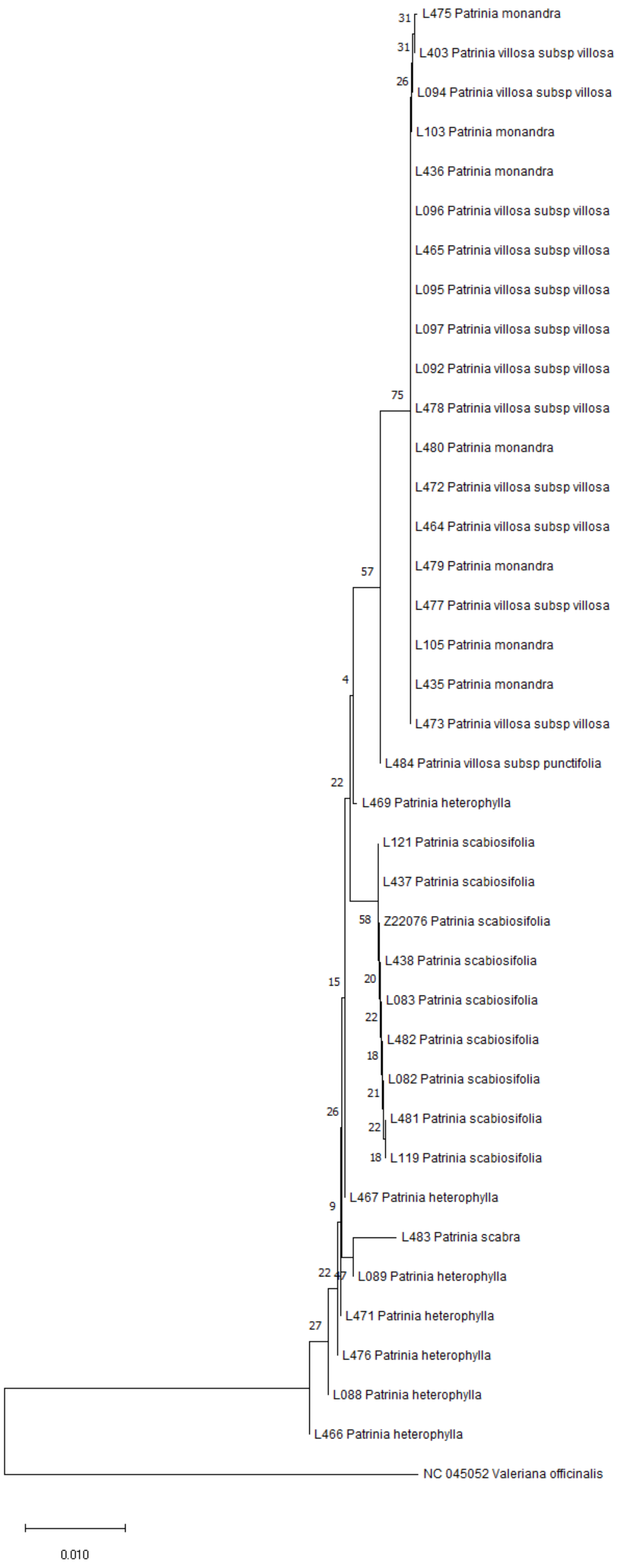

Fig. S7 – Neighbour-joining tree constructed from single DNA barcode region *rpl2-rpl23*

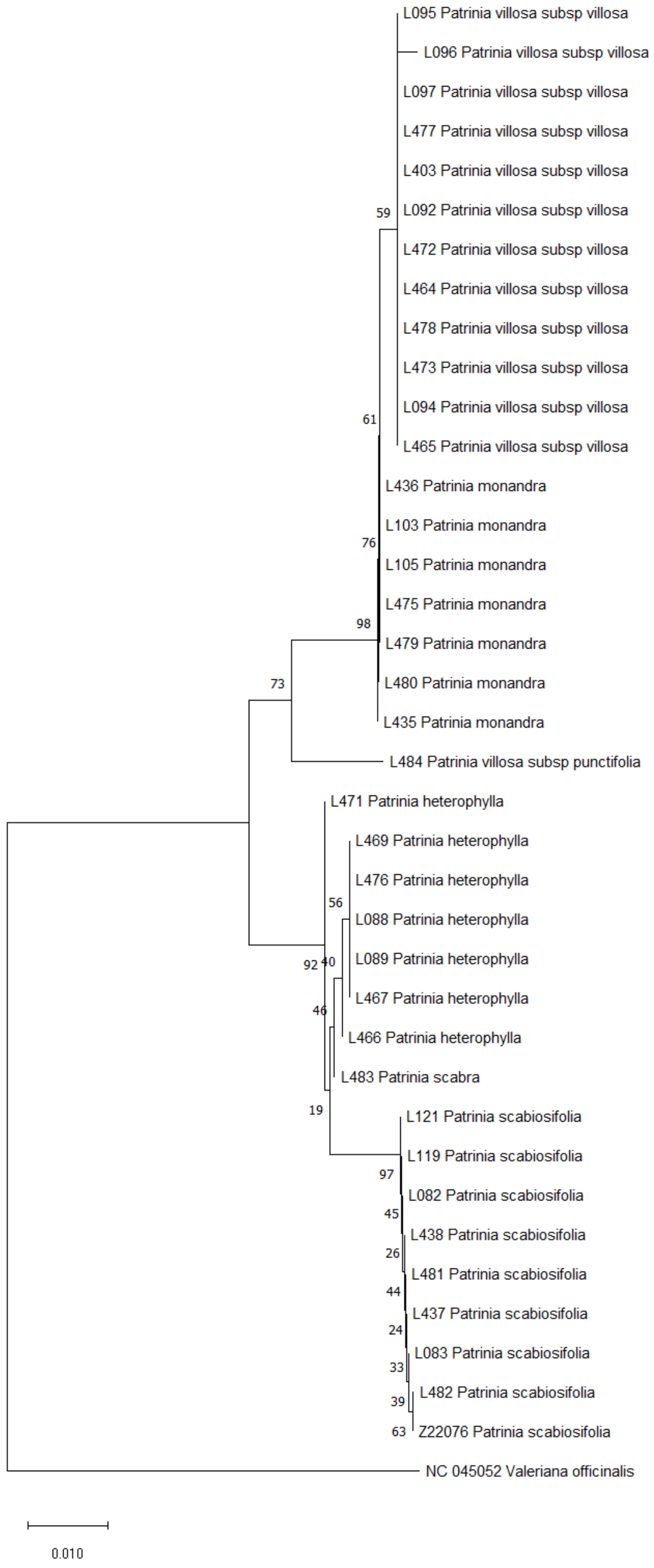

Fig. S8 – Neighbour-joining tree constructed from two-loci combination *atpB+petA*

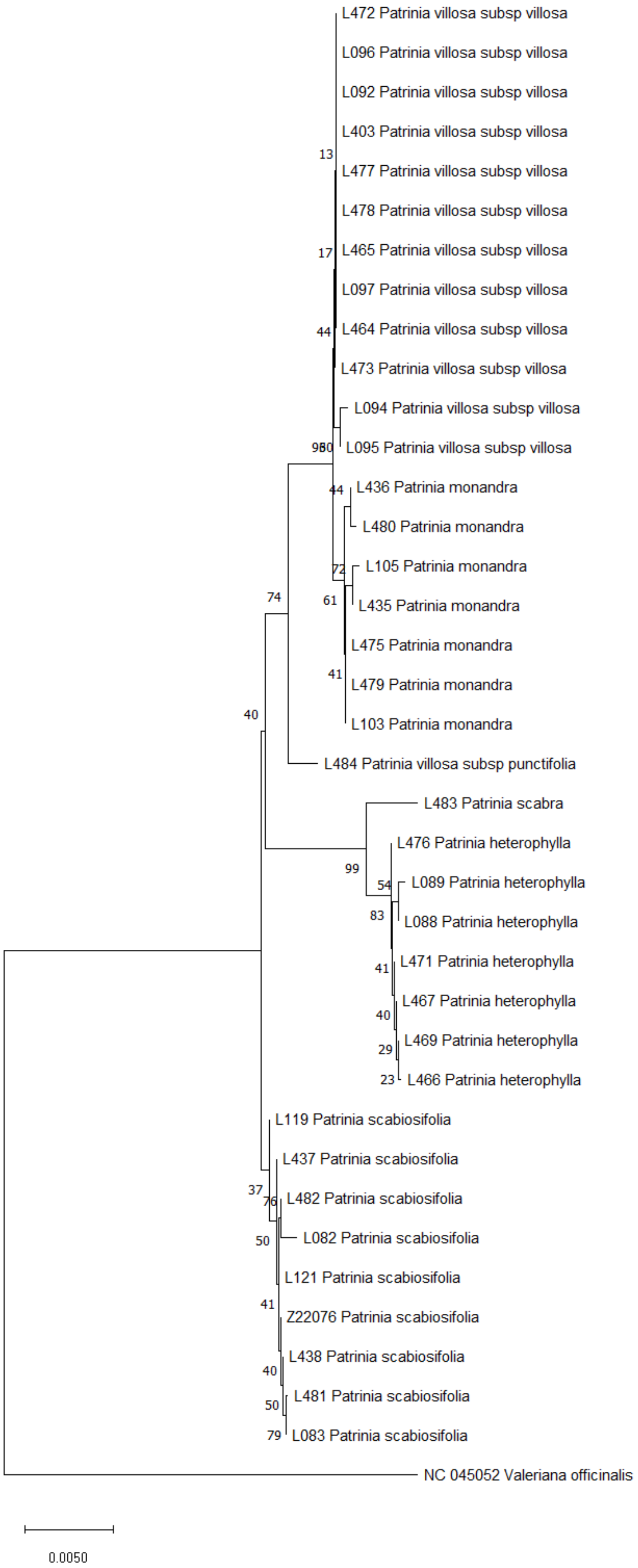

Fig. S9 – Neighbour-joining tree constructed from two-loci combination *petA+rpl2-rpl23*

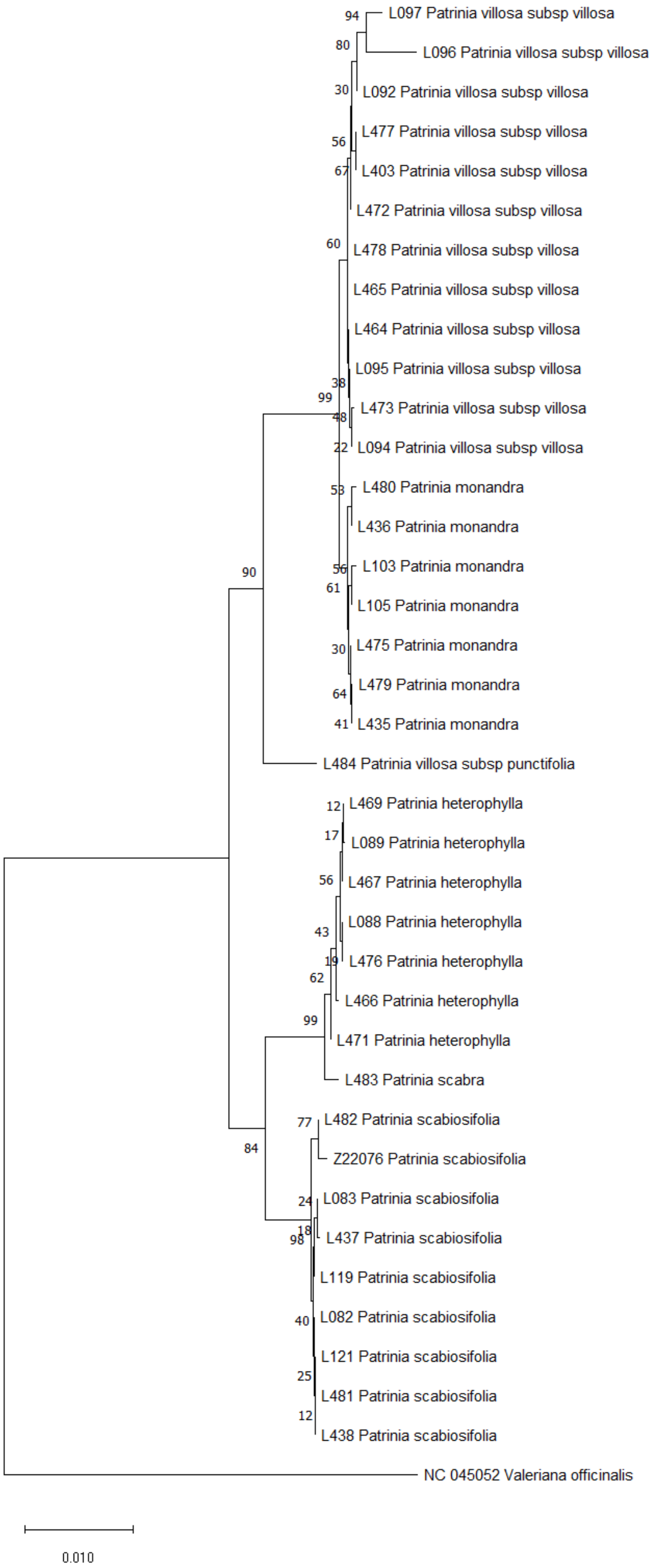

Fig. S10 – Neighbour-joining tree constructed from two-loci combination *petA+psaI-ycf4*

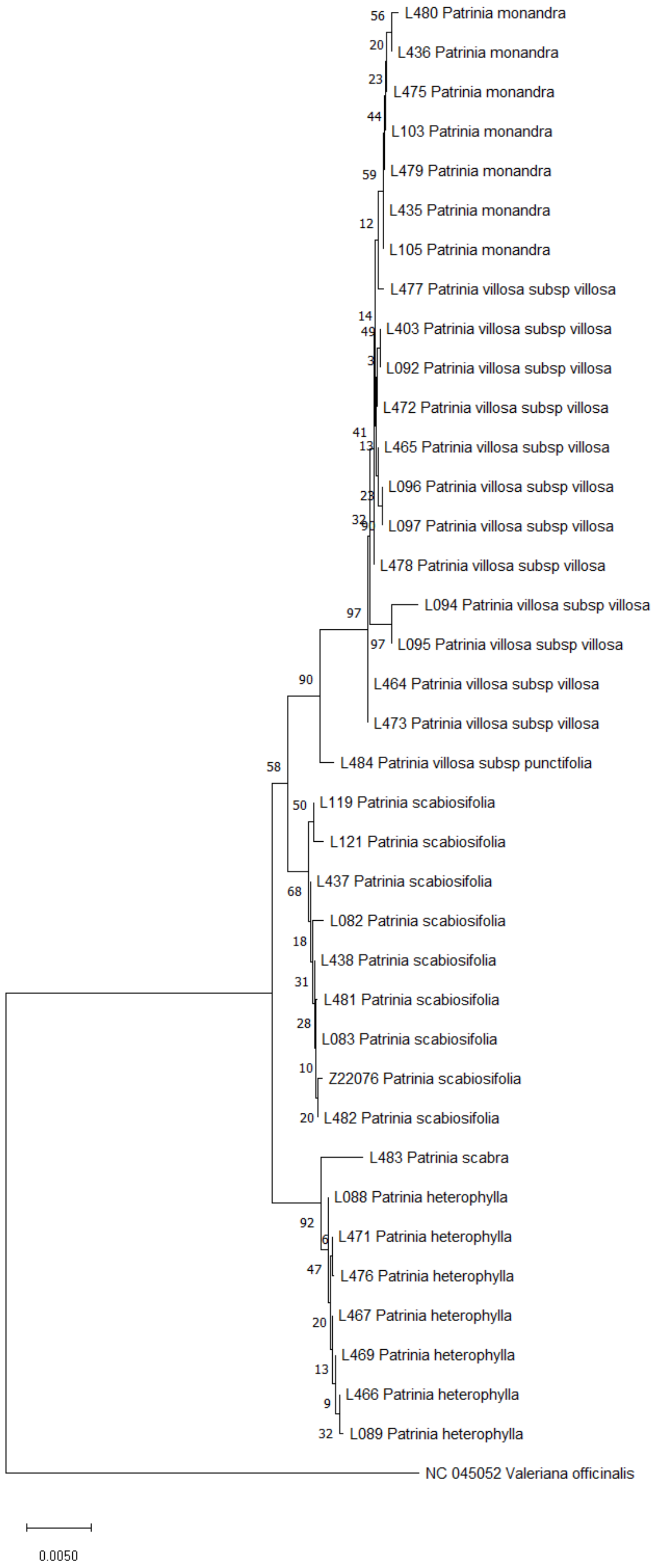

Fig. S11 – Neighbour-joining tree constructed from two-loci combination *atpB+psaI-ycf4*

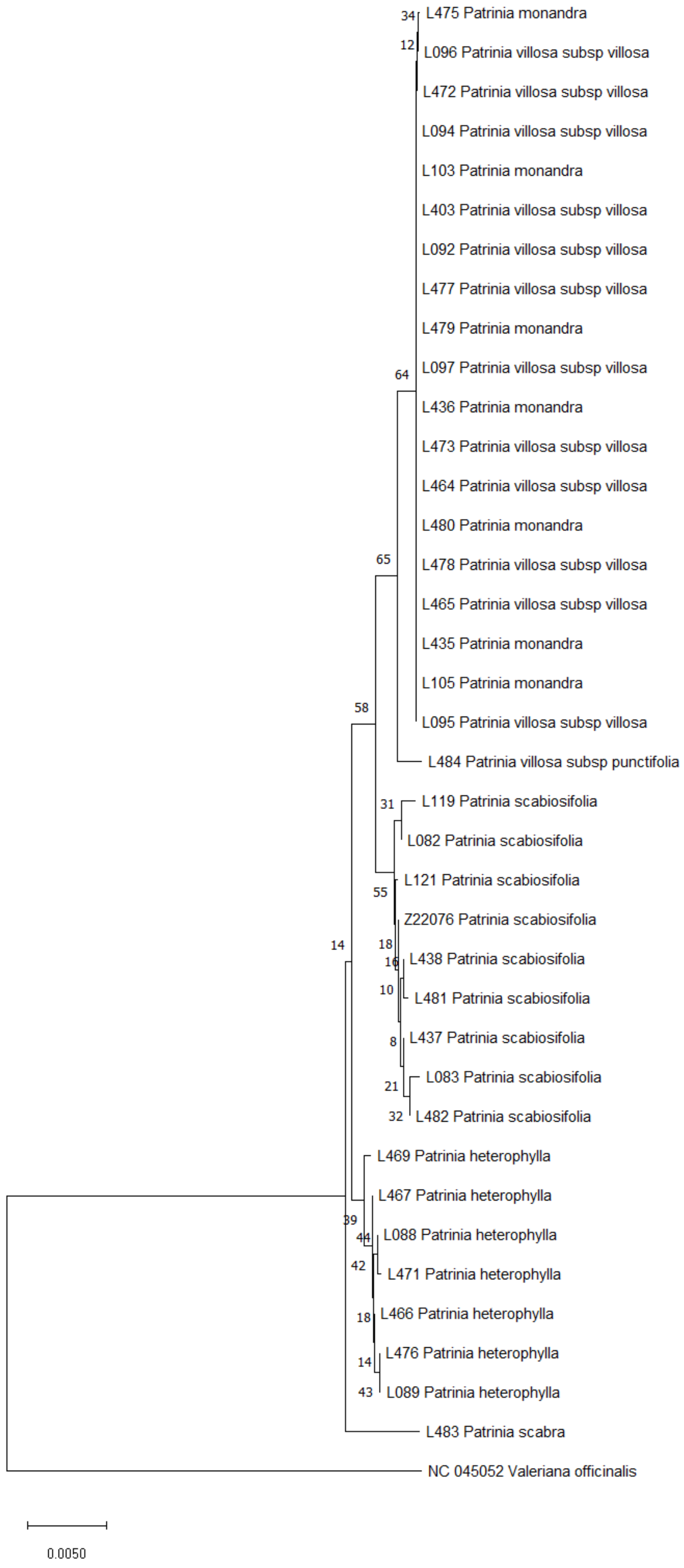

Fig. S12 – Neighbour-joining tree constructed from two-loci combination *atpB+rpl2-rpl23*

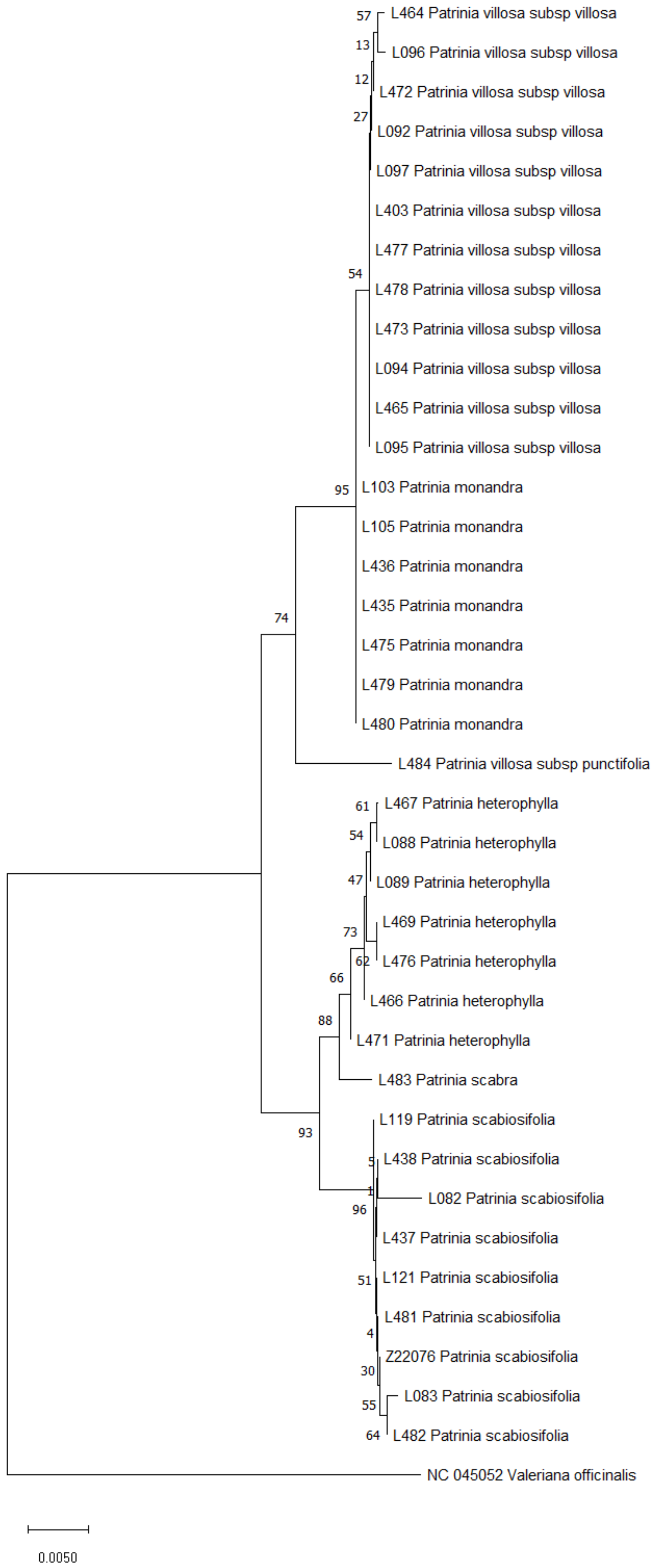

Fig. S13 – Neighbour-joining tree constructed from two-loci combination *psaI-ycf4+rpl2-rpl23*

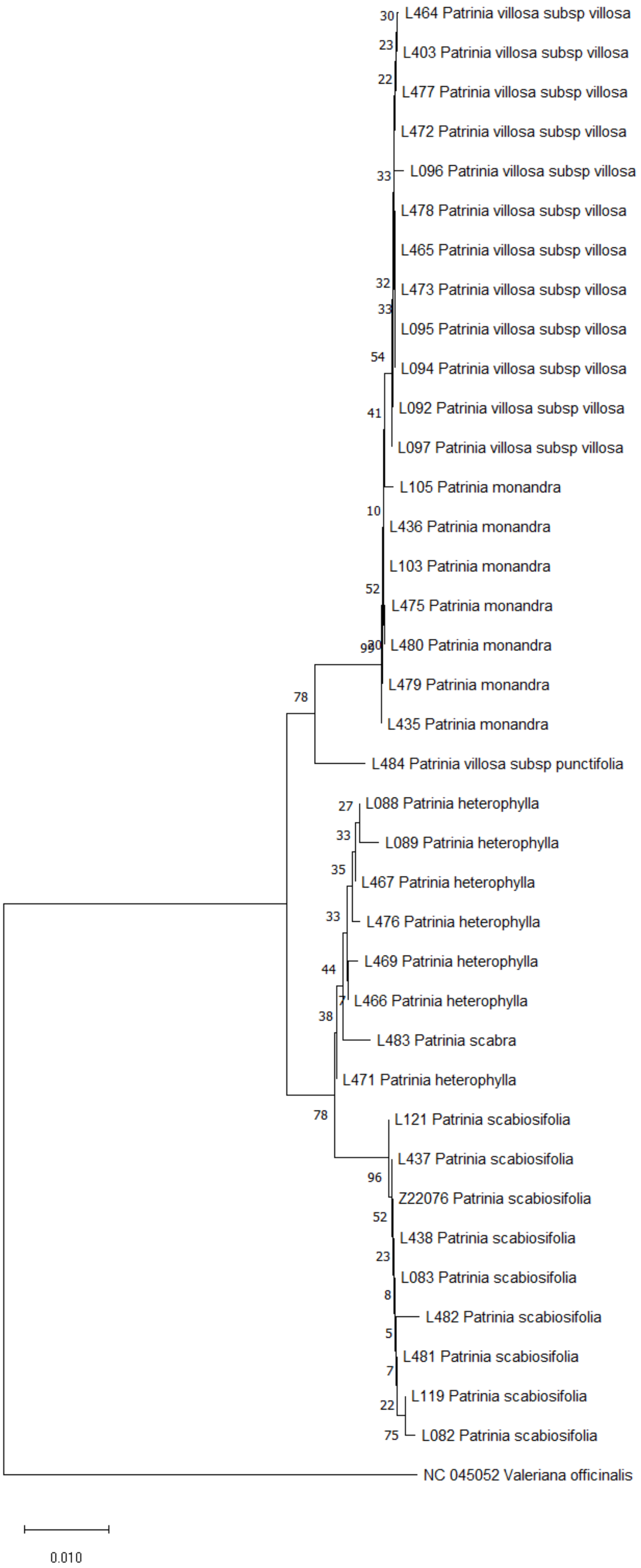

Fig. S14 – Neighbour-joining tree constructed from three-loci combination *petA+psaI-ycf4+rpl2-rpl23*

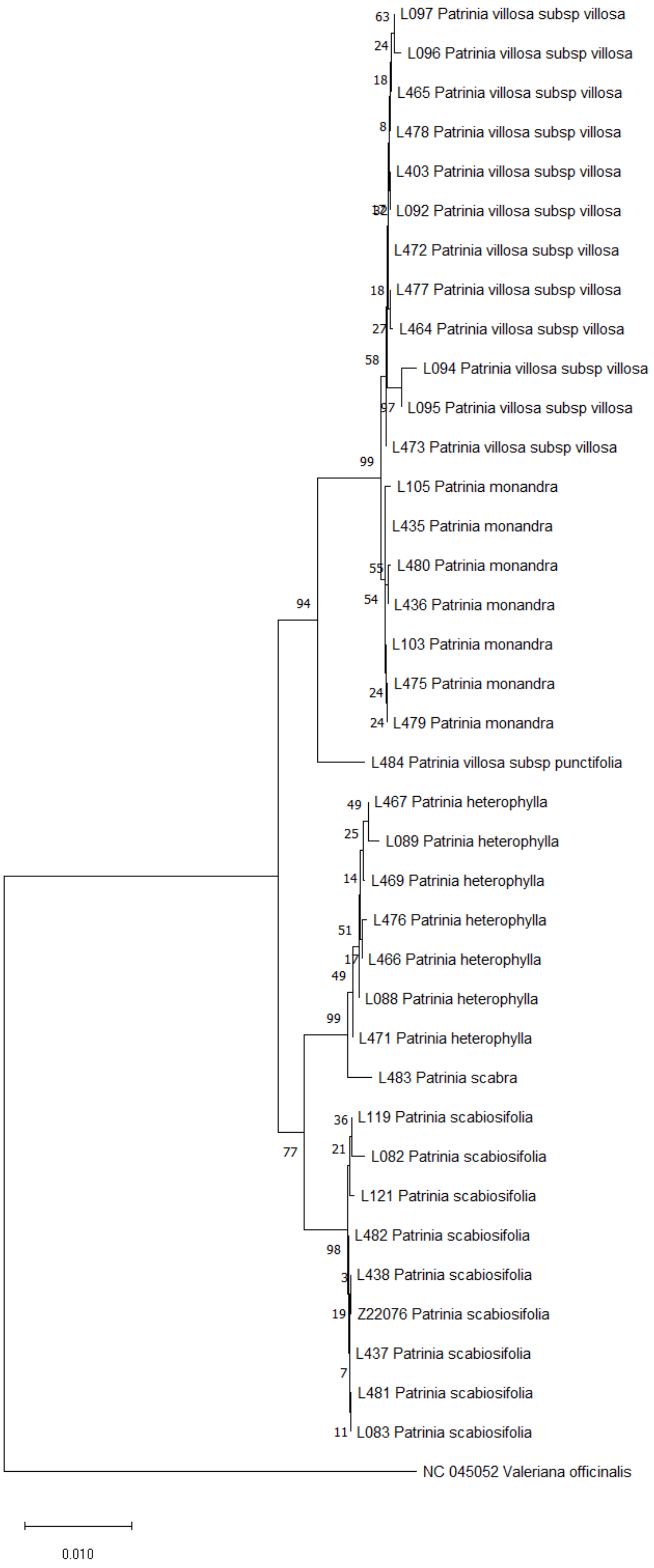

Fig. S15 – Neighbour-joining tree constructed from three-loci combination *atpB+petA+rpl2-rpl23*

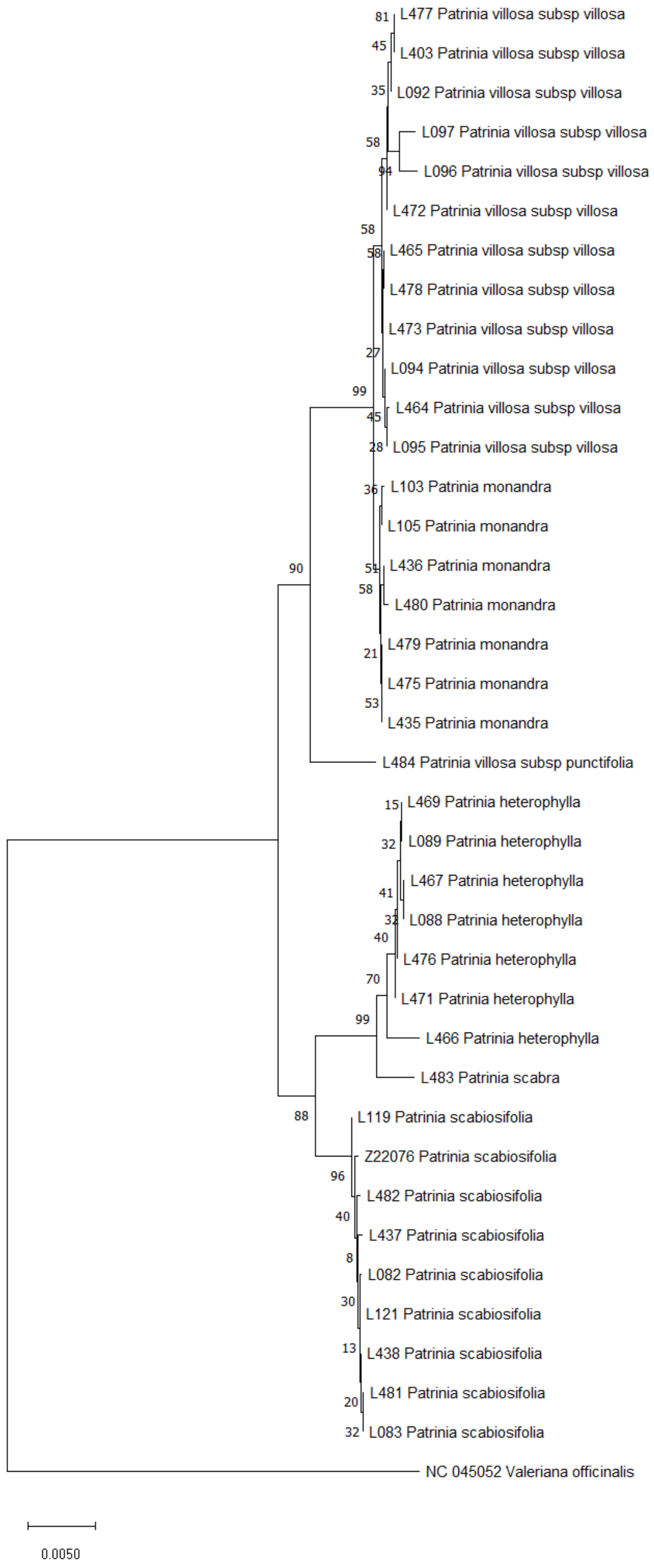

Fig. S16 – Neighbour-joining tree constructed from three-loci combination *atpB+petA+psaI-ycf4*

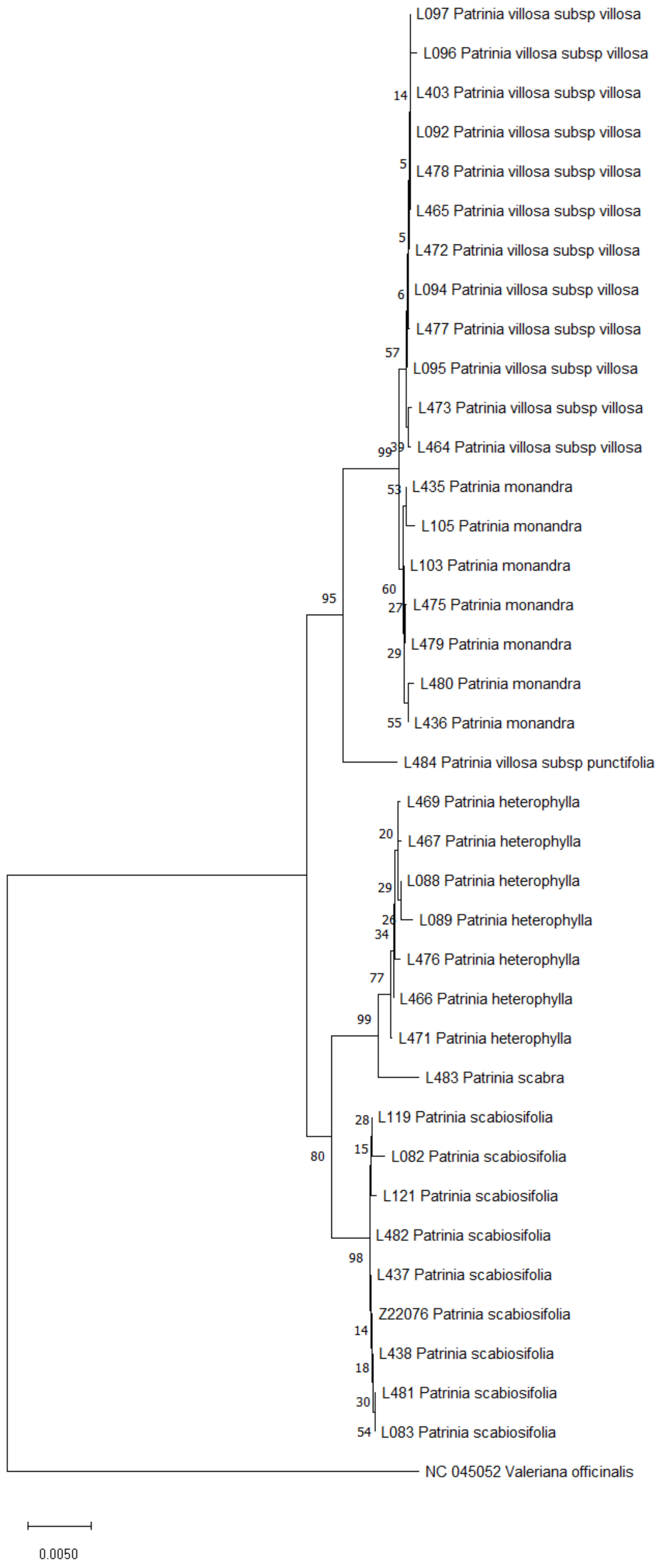

Fig. S17 – Neighbour-joining tree constructed from three-loci combination *atpB+psaI-ycf4+rpl2-rpl23*

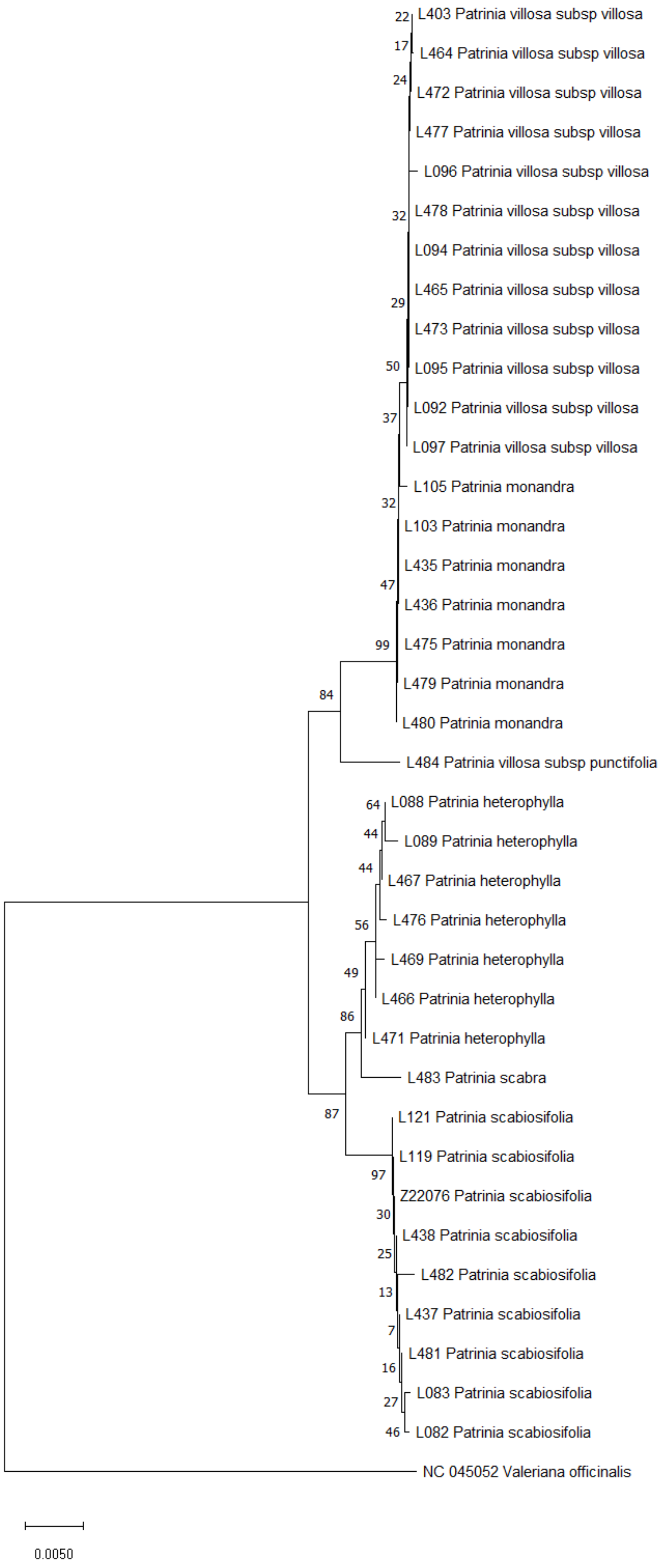

Fig. S18 – Neighbour-joining tree constructed from four-loci combination *atpB+petA+psaI-ycf4+rpl2-rpl23*

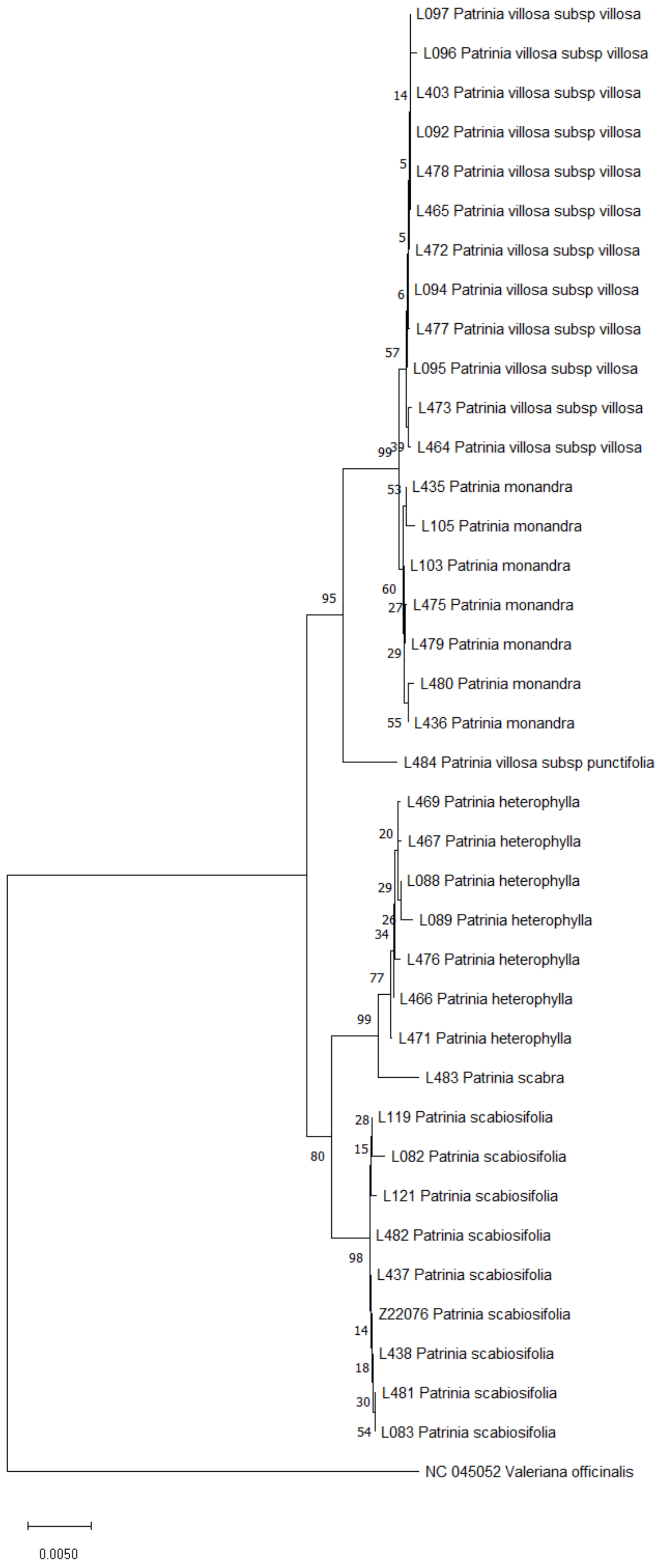

Fig. S19 – UPGMA tree constructed from single DNA barcode region *petA*

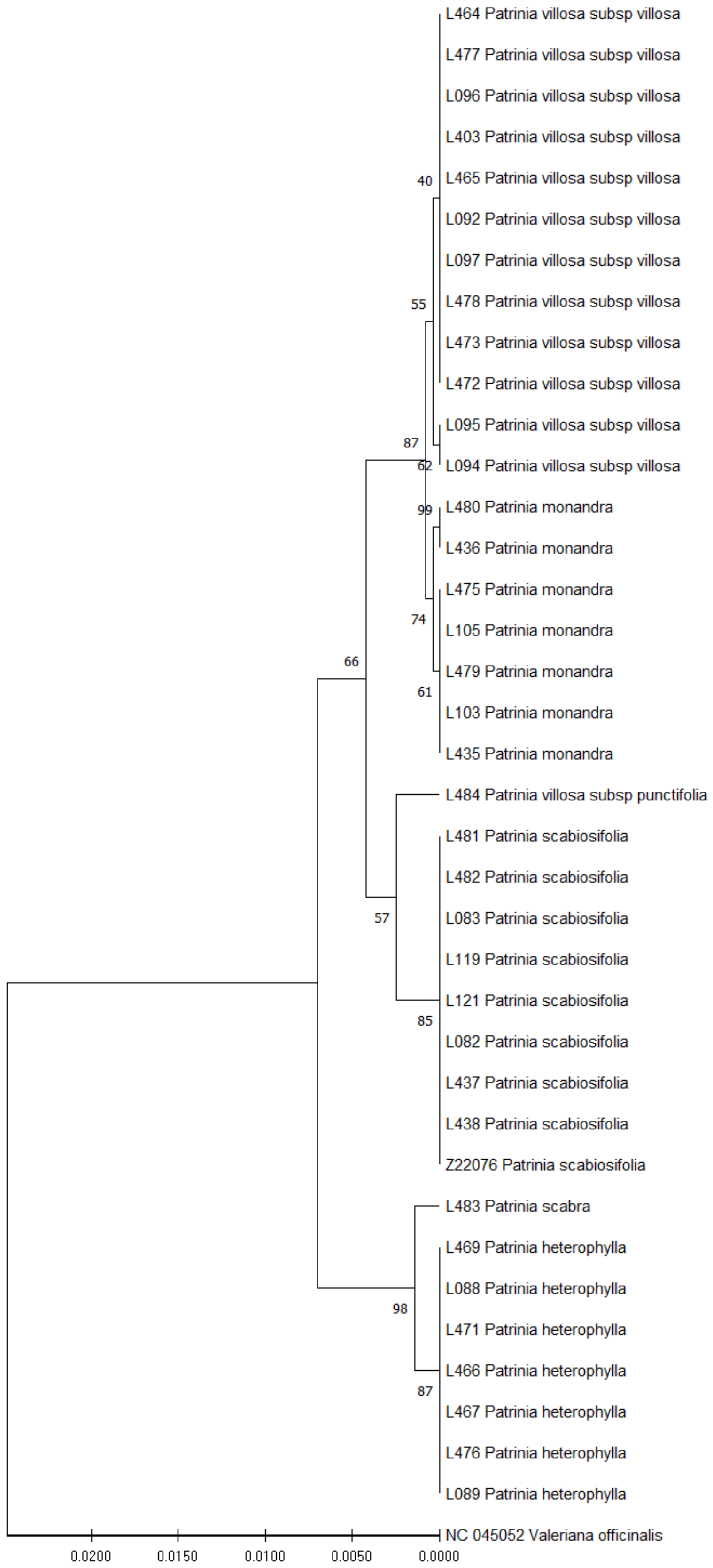

Fig. S20 – UPGMA tree constructed from single DNA barcode region *atpB*

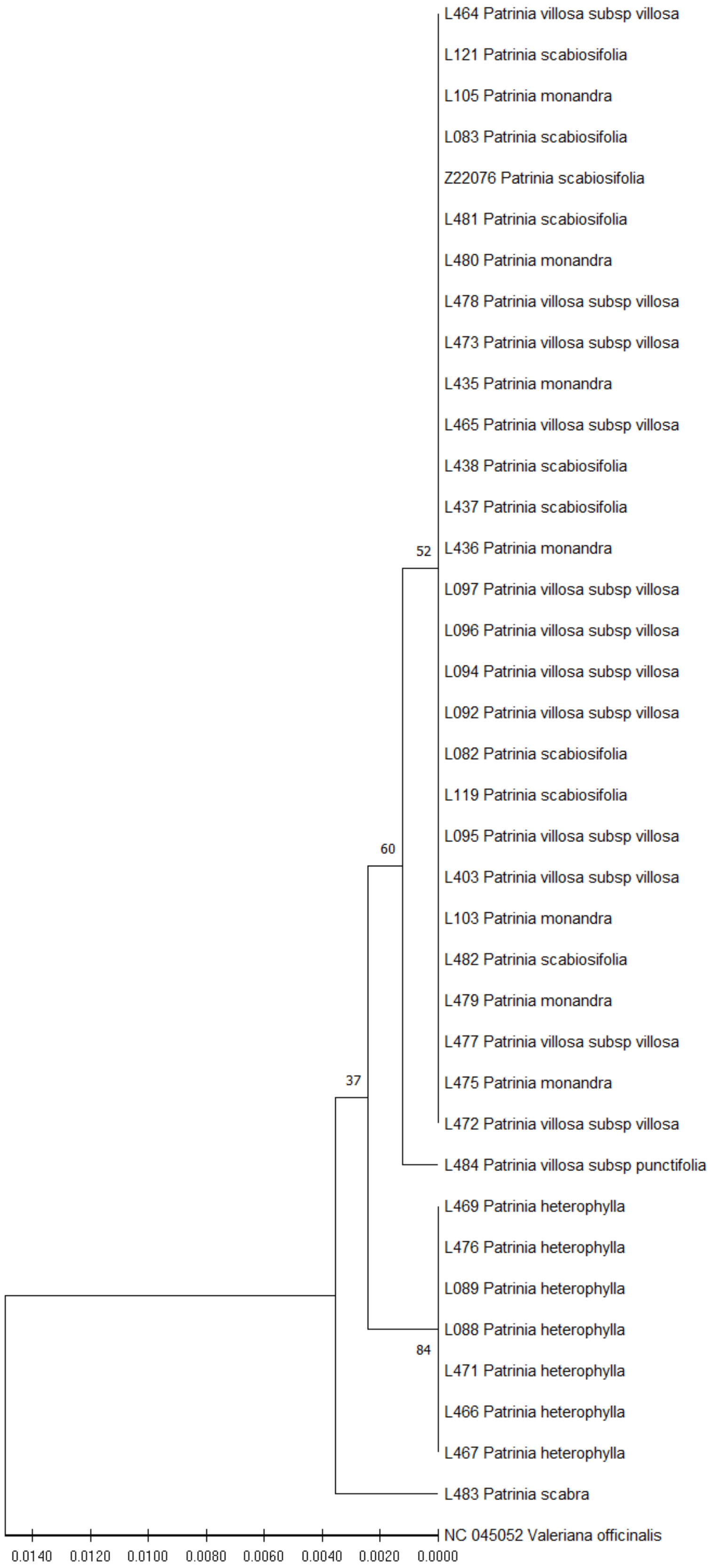

Fig. S21 – UPGMA tree constructed from single DNA barcode region *rpl2-rpl23*

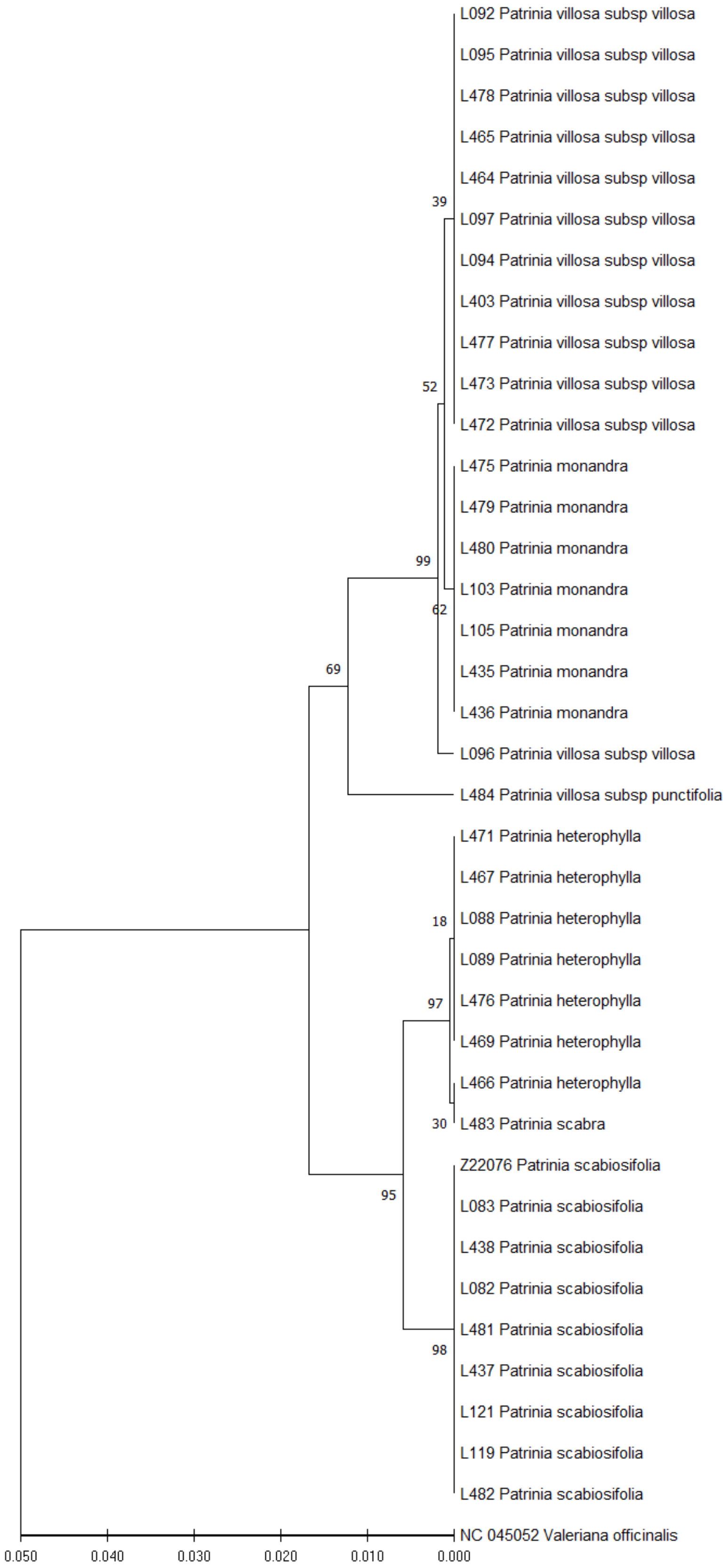

Fig. S22 – UPGMA tree constructed from single DNA barcode region *psaI-ycf4*

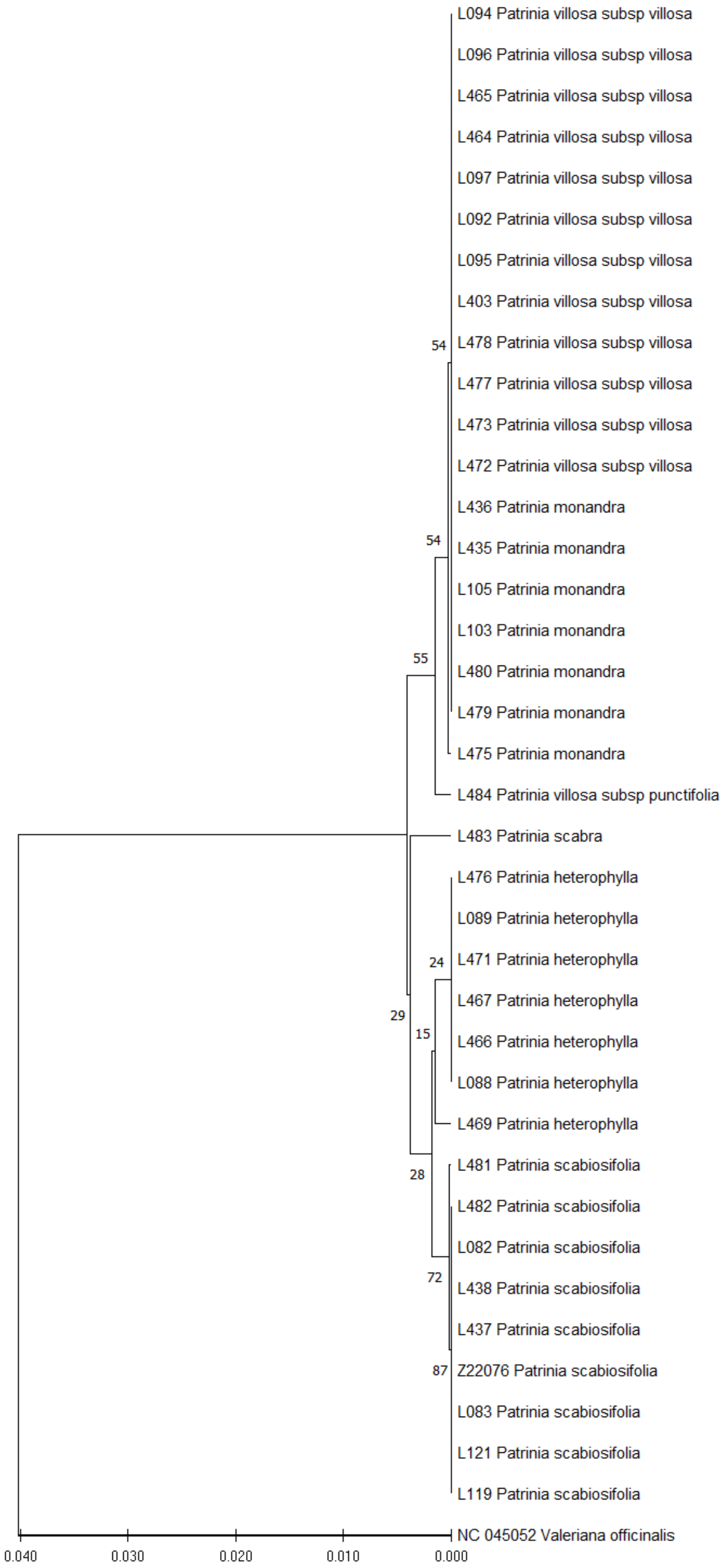

Fig. S23 – UPGMA tree constructed from four-loci combination *atpB+petA+psaI-ycf4+rpl2-rpl23*

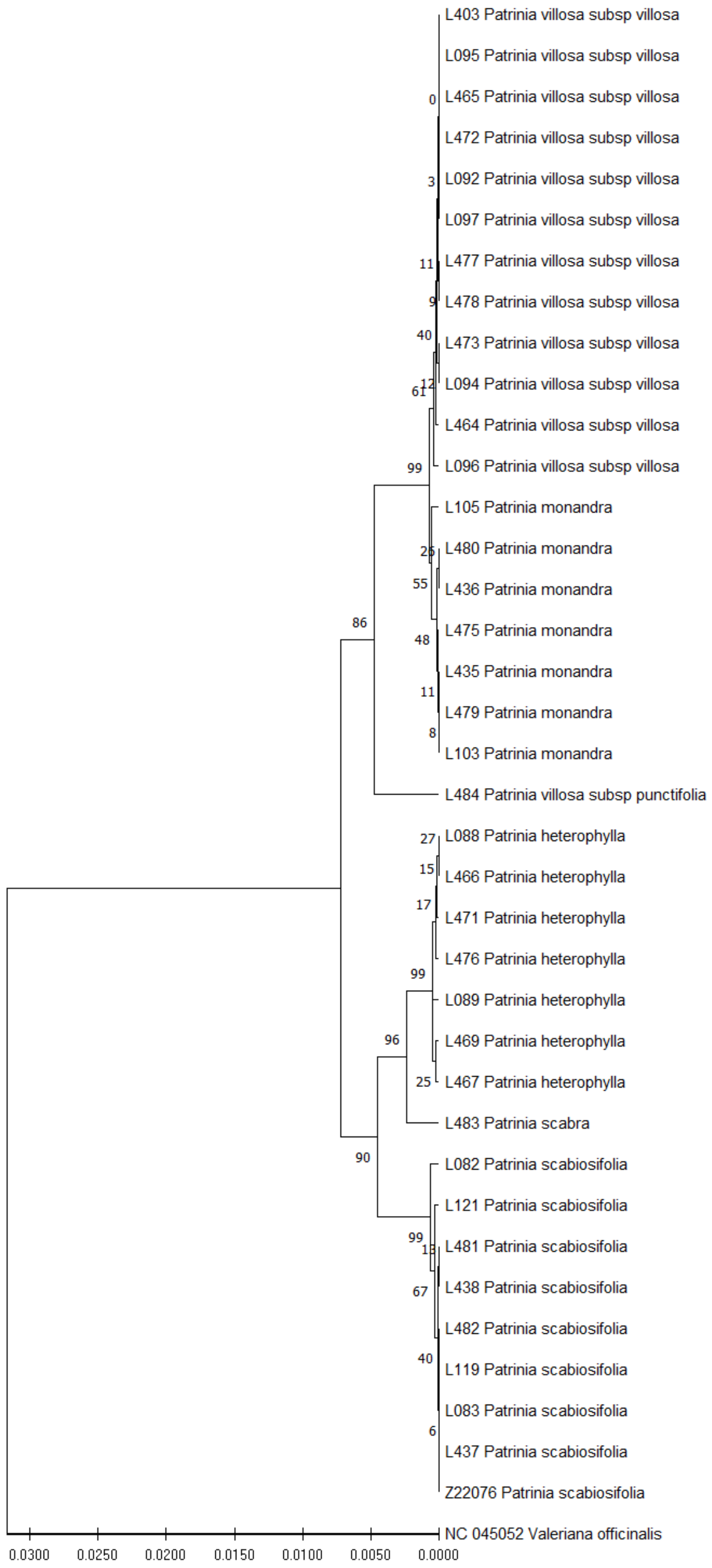

Fig. S24 – Maximum Likelihood tree constructed from single DNA barcode region *petA*

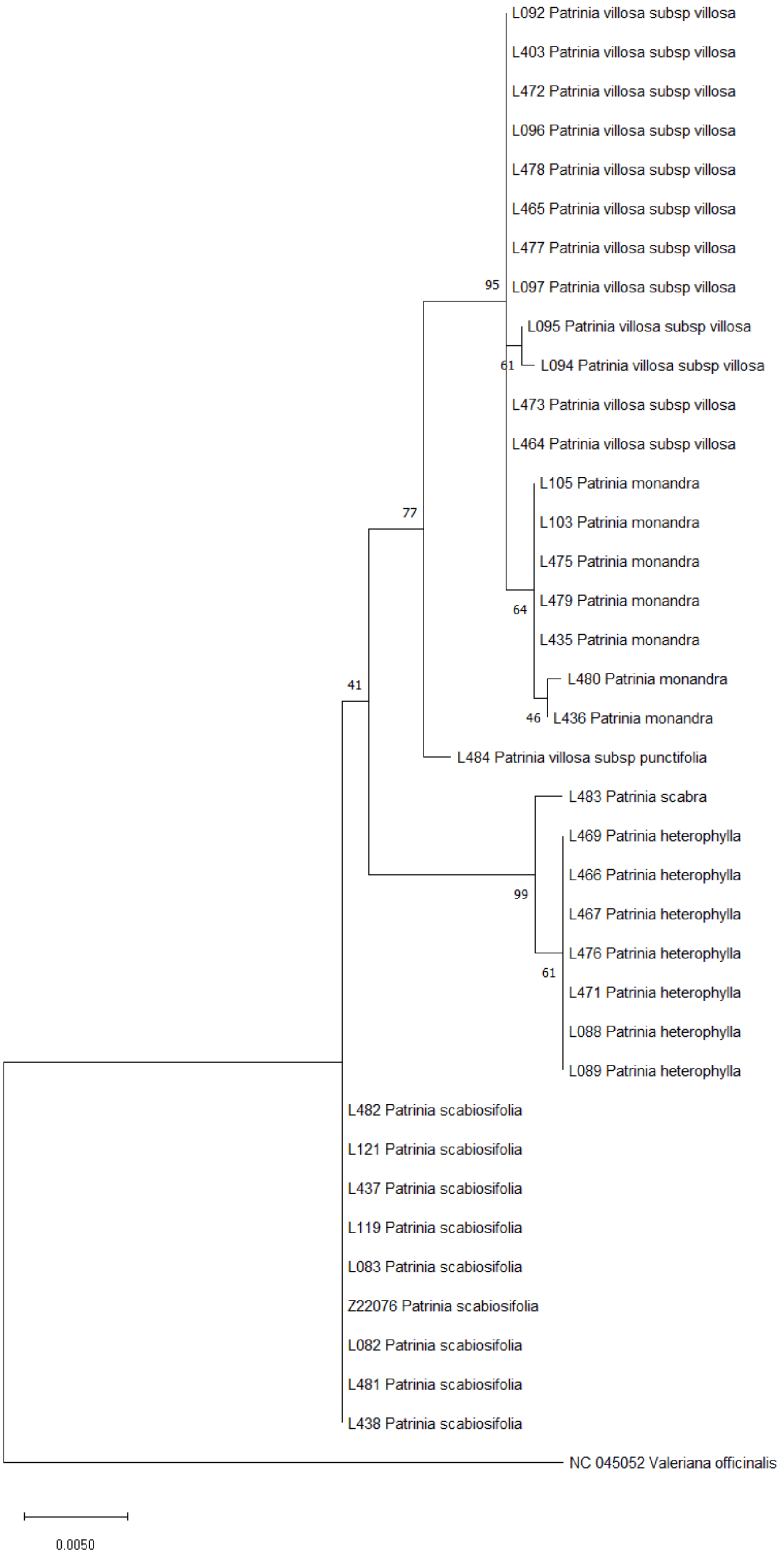

Fig. S25 – Maximum Likelihood tree constructed from single DNA barcode region *atpB*

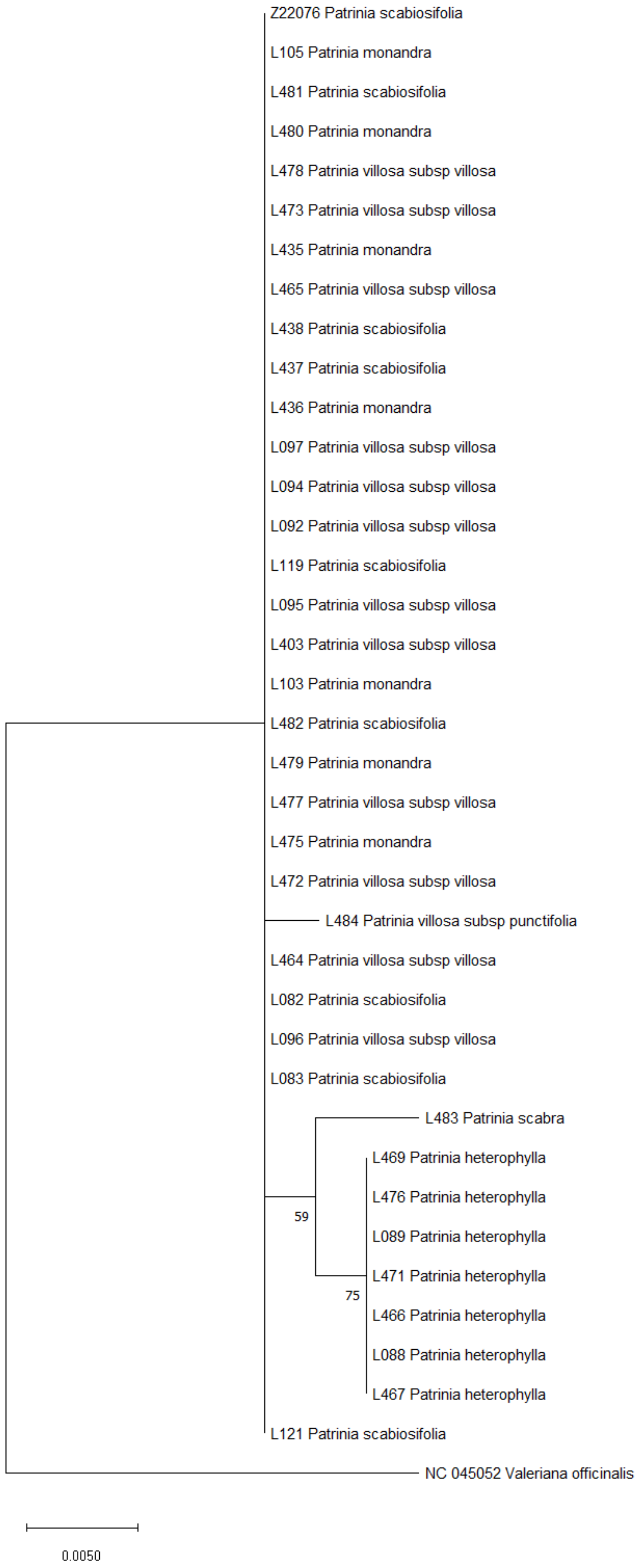

Fig. S26 – Maximum Likelihood tree constructed from single DNA barcode region *rpl2-rpl23*

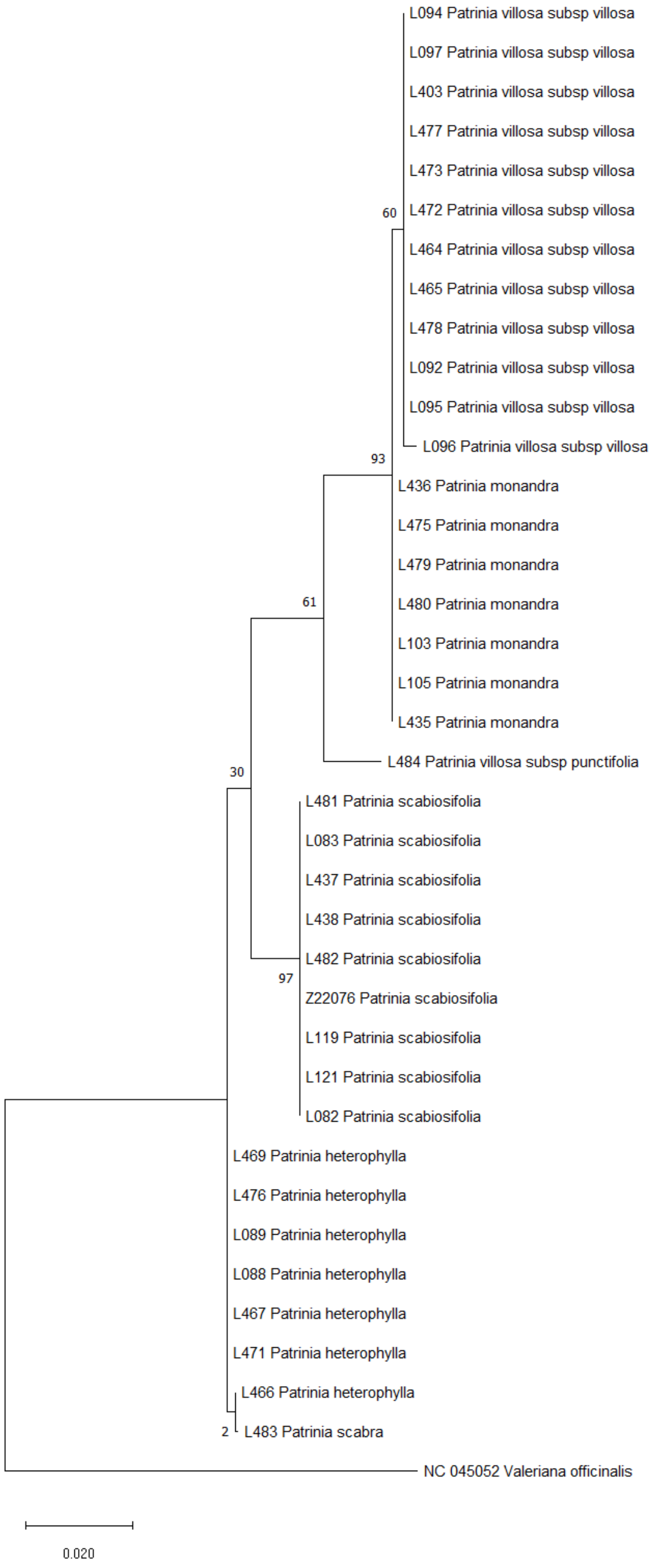

Fig. S27 – Maximum Likelihood tree constructed from single DNA barcode region *psaI-ycf4*

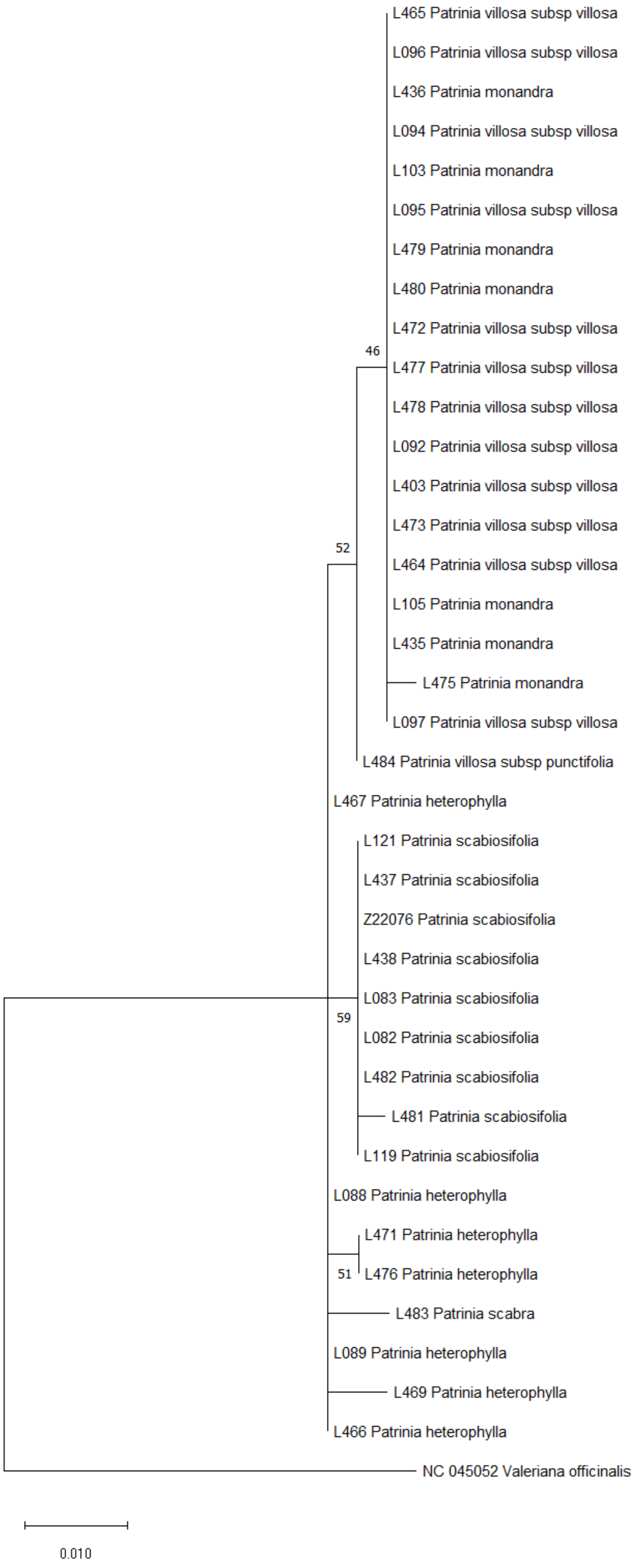

Fig. S28 – Maximum Likelihood tree constructed from four-loci combination *atpB+petA+psaI-ycf4+rpl2-rpl23*

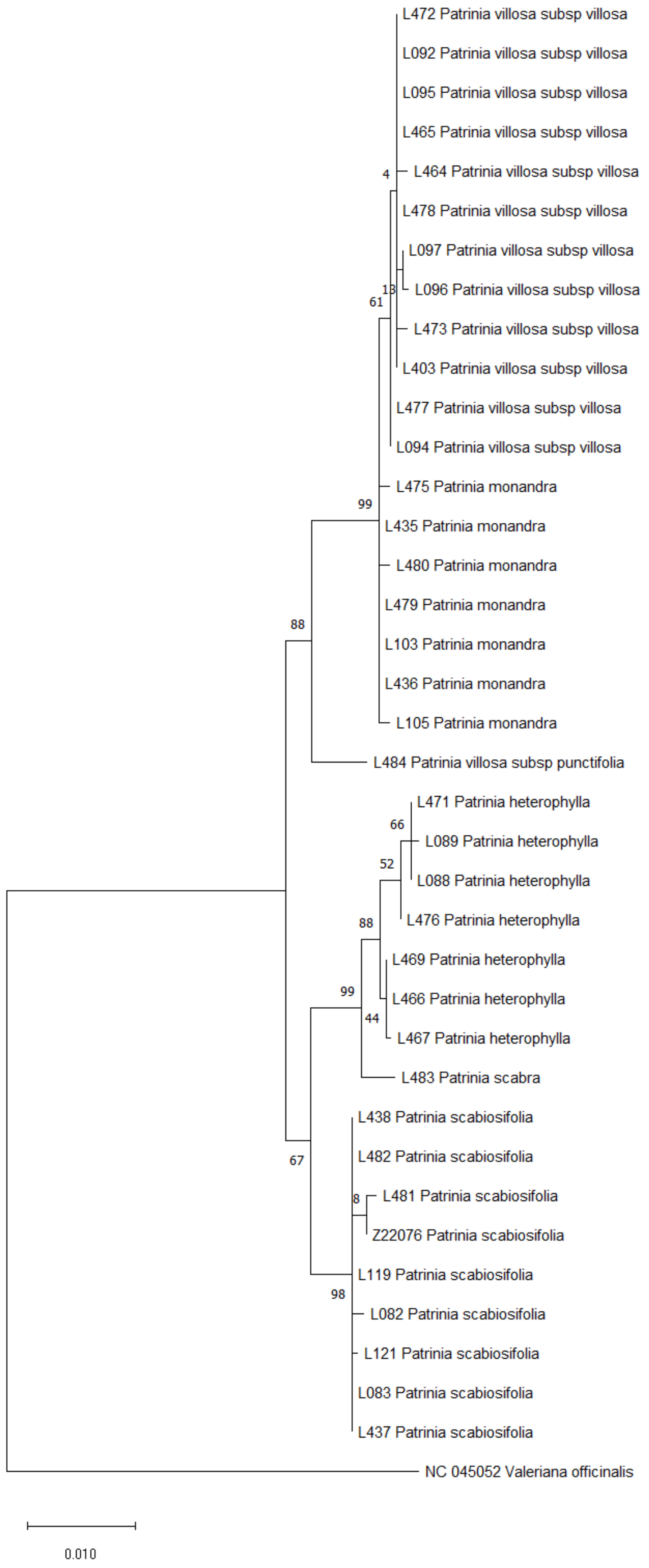

Fig. S29 – Gel electrophoresis of PCR amplification results of the region *petA*

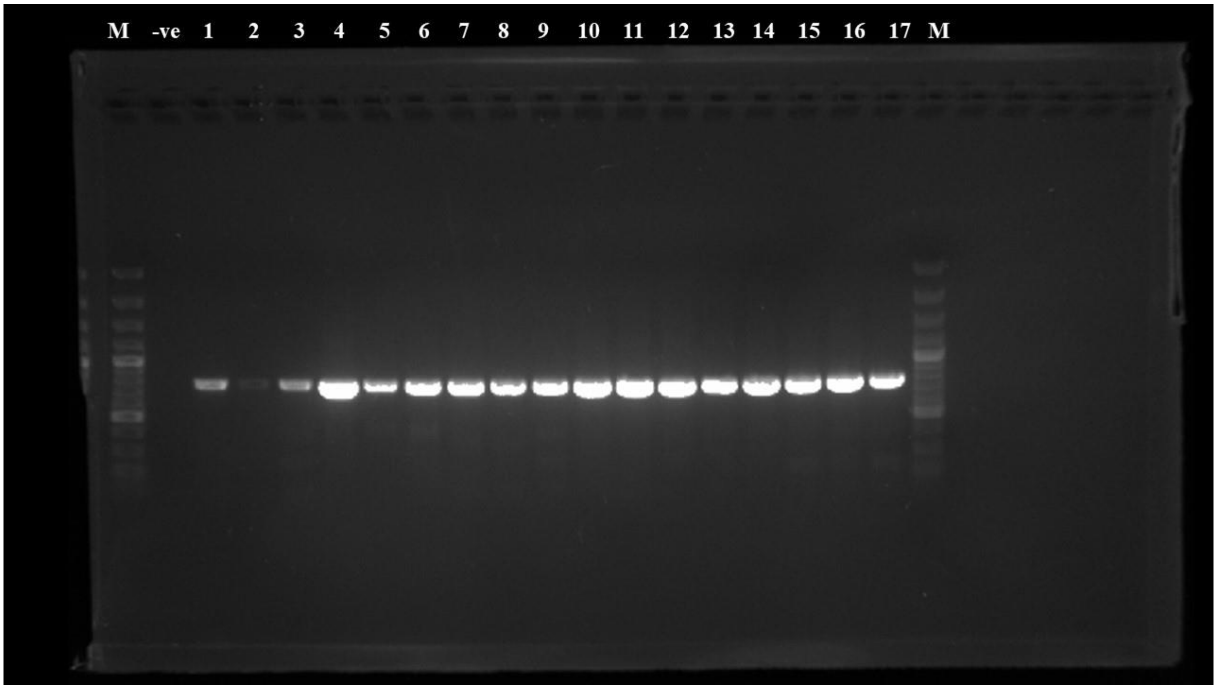

Lane annotation: M – 100 bp DNA ladder, -ve – negative control, 1 - M. C. Li 083, 2 - M. C. Li 089, 3 - M. C. Li 103, 4 - M. C. Li 403, 5 - M. C. Li 469, 6 - M. C. Li 471, 7 - M. C. Li 472, 8 - M. C. Li 473, 9 - M. C. Li 475, 10 - M. C. Li 476, 11- M. C. Li 477, 12 - M. C. Li 478, 13 - M. C. Li 479, 14 - M. C. Li 480, 15 - M. C. Li 481, 16 - M. C. Li 482, 17 - X. L. Zhao 22076.

Fig. S30 – Gel electrophoresis of PCR amplification results of the region *petA* (Continue)

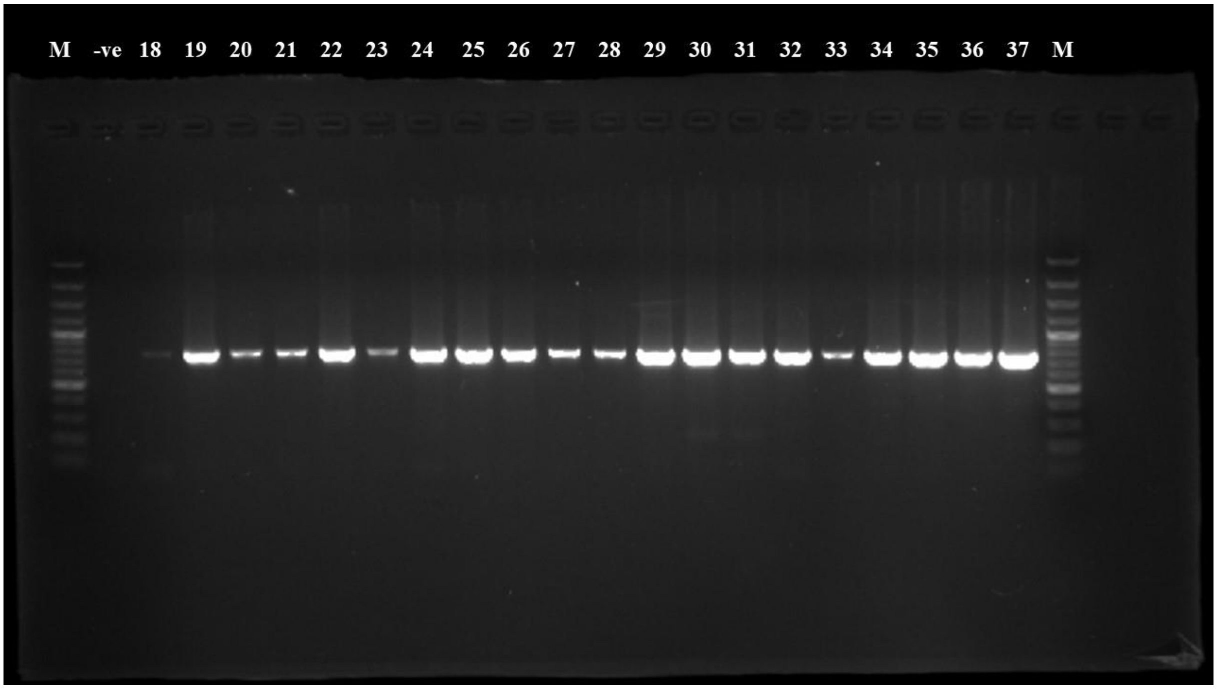

Lane annotation: M – 100 bp DNA ladder, -ve – negative control, 18 - M. C. Li 082, 19 - M. C. Li 088, 20 - M. C. Li 092, 21 - M. C. Li 094, 22 - M. C. Li 095, 23 - M. C. Li 096, 24 - M. C. Li 097, 25 - M. C. Li 105, 26 - M. C. Li 119, 27 - M. C. Li 120, 28 - M. C. Li 435, 29 - M. C. Li 436, 30 - M. C. Li 437, 31 - M. C. Li 438, 32 - M. C. Li 464, 33 - M. C. Li 465, 34 - M. C. Li 466, 35 - M. C. Li 467, 36 - M. C. Li 483, 37 - M. C. Li 484.

Fig. S31 – Gel electrophoresis of PCR amplification results of the region *atpB*

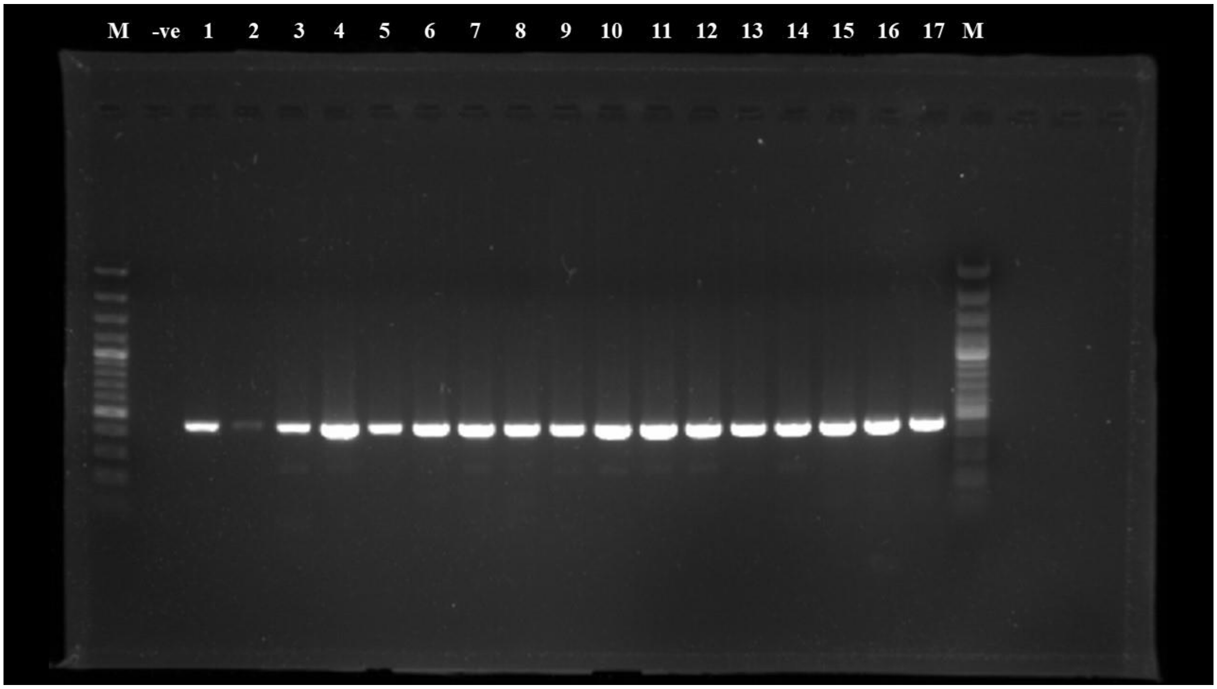

Lane annotation: M – 100 bp DNA ladder, -ve – negative control, 1 - M. C. Li 083, 2 - M. C. Li 089, 3 - M. C. Li 103, 4 - M. C. Li 403, 5 - M. C. Li 469, 6 - M. C. Li 471, 7 - M. C. Li 472, 8 - M. C. Li 473, 9 - M. C. Li 475, 10 - M. C. Li 476, 11- M. C. Li 477, 12 - M. C. Li 478, 13 - M. C. Li 479, 14 - M. C. Li 480, 15 - M. C. Li 481, 16 - M. C. Li 482, 17 - X. L. Zhao 22076.

Fig. S32 – Gel electrophoresis of PCR amplification results of the region *atpB* (Continue)

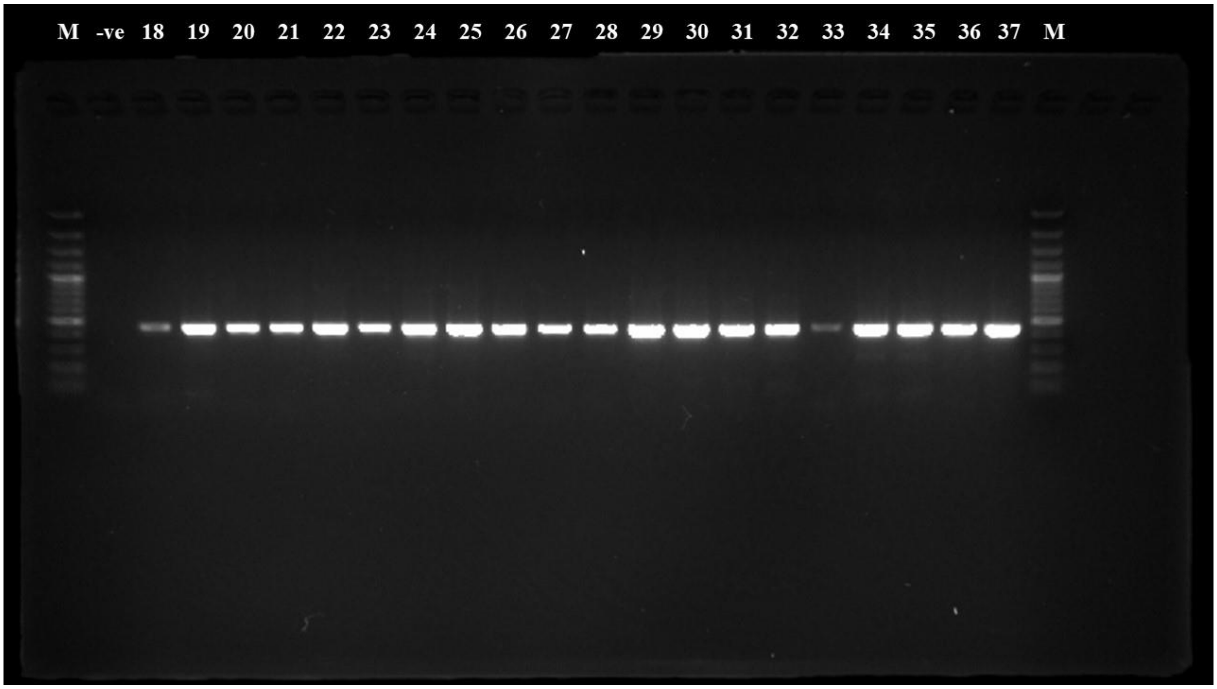

Lane annotation: M – 100 bp DNA ladder, -ve – negative control, 18 - M. C. Li 082, 19 - M. C. Li 088, 20 - M. C. Li 092, 21 - M. C. Li 094, 22 - M. C. Li 095, 23 - M. C. Li 096, 24 - M. C. Li 097, 25 - M. C. Li 105, 26 - M. C. Li 119, 27 - M. C. Li 120, 28 - M. C. Li 435, 29 - M. C. Li 436, 30 - M. C. Li 437, 31 - M. C. Li 438, 32 - M. C. Li 464, 33 - M. C. Li 465, 34 - M. C. Li 466, 35 - M. C. Li 467, 36 - M. C. Li 483, 37 - M. C. Li 484.

Fig. S33 – Gel electrophoresis of PCR amplification results of the region *rpl2-rpl23*

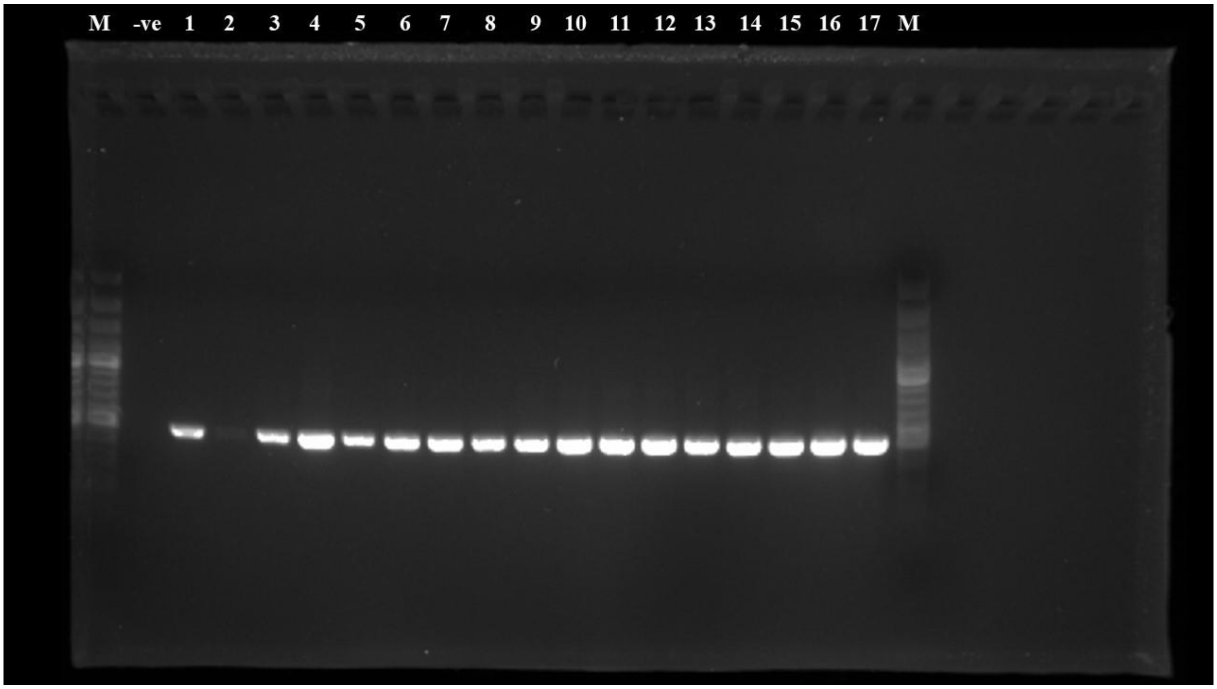

Lane annotation: M – 100 bp DNA ladder, -ve – negative control, 1 - M. C. Li 083, 2 - M. C. Li 089, 3 - M. C. Li 103, 4 - M. C. Li 403, 5 - M. C. Li 469, 6 - M. C. Li 471, 7 - M. C. Li 472, 8 - M. C. Li 473, 9 - M. C. Li 475, 10 - M. C. Li 476, 11- M. C. Li 477, 12 - M. C. Li 478, 13 - M. C. Li 479, 14 - M. C. Li 480, 15 - M. C. Li 481, 16 - M. C. Li 482, 17 - X. L. Zhao 22076.

Fig. S34 – Gel electrophoresis of PCR amplification results of the region *rpl2-rpl23* (Continue)

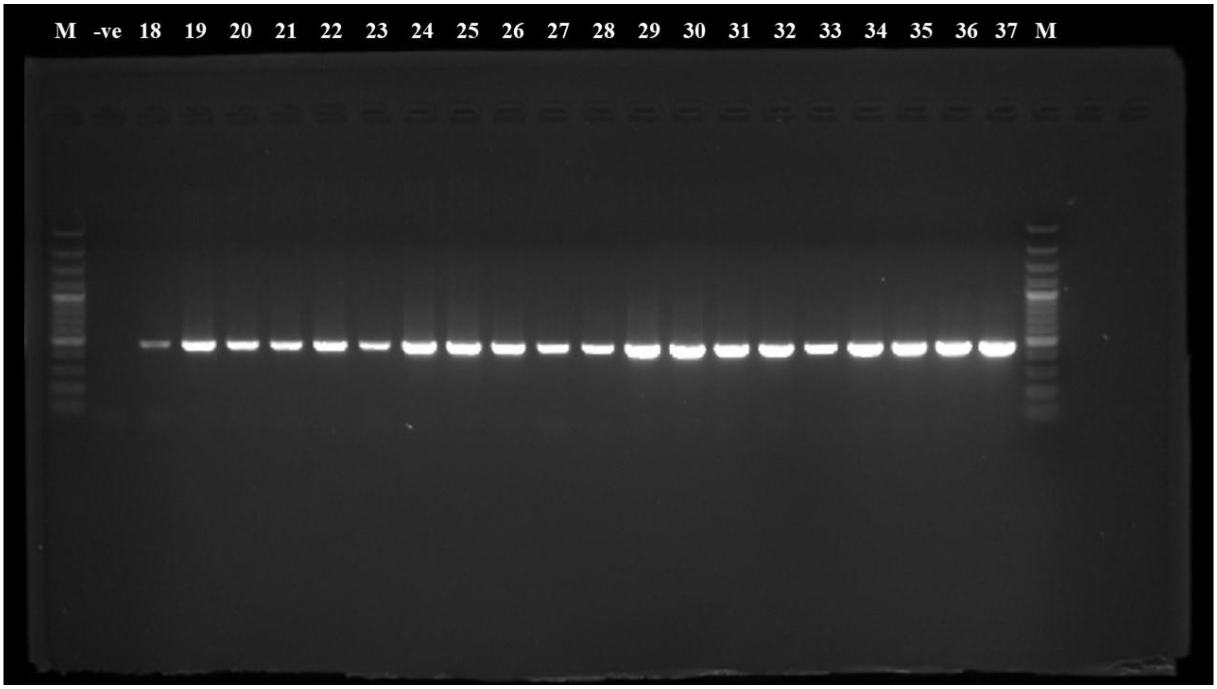

Lane annotation: M – 100 bp DNA ladder, -ve – negative control, 18 - M. C. Li 082, 19 - M. C. Li 088, 20 - M. C. Li 092, 21 - M. C. Li 094, 22 - M. C. Li 095, 23 - M. C. Li 096, 24 - M. C. Li 097, 25 - M. C. Li 105, 26 - M. C. Li 119, 27 - M. C. Li 120, 28 - M. C. Li 435, 29 - M. C. Li 436, 30 - M. C. Li 437, 31 - M. C. Li 438, 32 - M. C. Li 464, 33 - M. C. Li 465, 34 - M. C. Li 466, 35 - M. C. Li 467, 36 - M. C. Li 483, 37 - M. C. Li 484.

Fig. S35 – Gel electrophoresis of PCR amplification results of the region *psaI-ycf4*

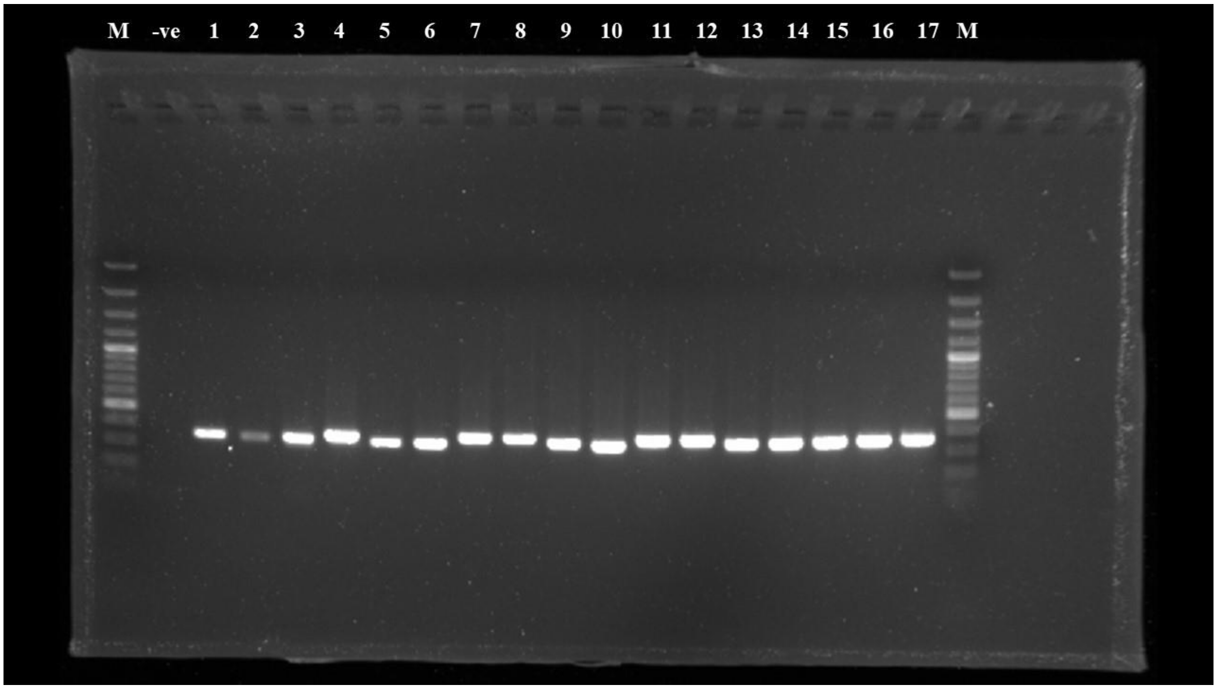

Lane annotation: M – 100 bp DNA ladder, -ve – negative control, 1 - M. C. Li 083, 2 - M. C. Li 089, 3 - M. C. Li 103, 4 - M. C. Li 403, 5 - M. C. Li 469, 6 - M. C. Li 471, 7 - M. C. Li 472, 8 - M. C. Li 473, 9 - M. C. Li 475, 10 - M. C. Li 476, 11- M. C. Li 477, 12 - M. C. Li 478, 13 - M. C. Li 479, 14 - M. C. Li 480, 15 - M. C. Li 481, 16 - M. C. Li 482, 17 - X. L. Zhao 22076.

Fig. S36 – Gel electrophoresis of PCR amplification results of the region *psaI-ycf4* (Continue)

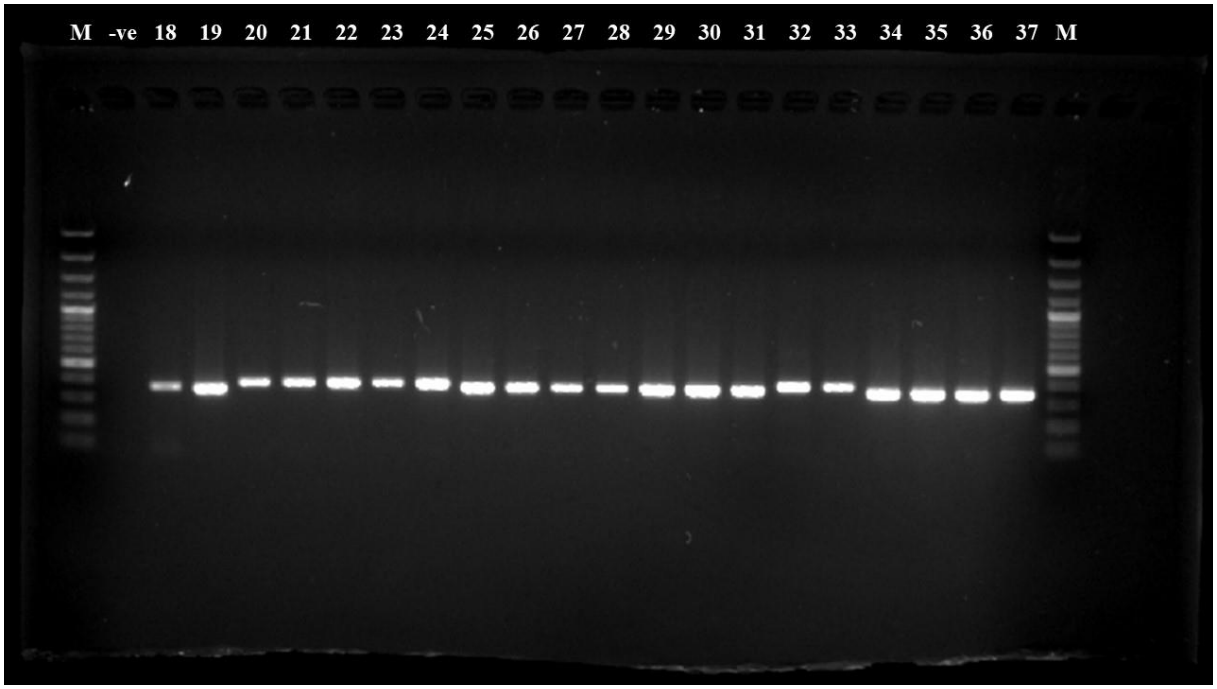

Lane annotation: M – 100 bp DNA ladder, -ve – negative control, 18 - M. C. Li 082, 19 - M. C. Li 088, 20 - M. C. Li 092, 21 - M. C. Li 094, 22 - M. C. Li 095, 23 - M. C. Li 096, 24 - M. C. Li 097, 25 - M. C. Li 105, 26 - M. C. Li 119, 27 - M. C. Li 120, 28 - M. C. Li 435, 29 - M. C. Li 436, 30 - M. C. Li 437, 31 - M. C. Li 438, 32 - M. C. Li 464, 33 - M. C. Li 465, 34 - M. C. Li 466, 35 - M. C. Li 467, 36 - M. C. Li 483, 37 - M. C. Li 484.

Table S1 – Complete chloroplast genomes used for alignment and sliding window analysis

No.	NCBI Accession no.	Species	Genome size (bp)
1	NC_060729.1	*Patrinia rupestris*	159,266
2	NC_061006.1	*Patrinia monandra*	158,940
3	NC_042190.1	*Patrinia villosa*	158,922
4	NC_042178.1	*Patrinia scabiosifolia*	158,872
5	NC_036835.1	*Patrinia saniculifolia*	153,775
6	NC_045047.1	*Patrinia heterophylla*	151,964
7	NC_045048.1	*Patrinia scabra*	151,267
8	AP017910.1	*Patrinia triloba* var. *takeuchiana*	158,524
9	MN524609.1	*Patrinia scabiosifolia*	154,019

Table S2 – Information on the total genomic DNA extracted from the studied samples

Code	Collector no.	Species	Sampled size (g)	Nucleic Acid (ng/uL)	A260	A260/A280
L082	M. C. Li 082	*Patrinia scabiosifolia* Link	0.0517	24.3	0.485	1.12
L083	M. C. Li 083	*Patrinia scabiosifolia* Link	0.0584	49.0	0.981	1.52
L088	M. C. Li 088	*Patrinia heterophylla* Bunge	0.0240	29.7	0.594	1.47
L089	M. C. Li 089	*Patrinia heterophylla* Bunge	0.0578	47.0	0.940	1.26
L092	M. C. Li 092	*Patrinia villosa* (Thunb.) Juss. subsp. *villosa*	0.0515	15.5	0.310	1.25
L094	M. C. Li 094	*Patrinia villosa* (Thunb.) Juss. subsp. *villosa*	0.0512	25.1	0.501	1.5
L095	M. C. Li 095	*Patrinia villosa* (Thunb.) Juss. subsp. *villosa*	0.0542	20.1	0.403	1.12
L096	M. C. Li 096	*Patrinia villosa* (Thunb.) Juss. subsp. *villosa*	0.0527	37.0	0.739	1.58
L097	M. C. Li 097	*Patrinia villosa* (Thunb.) Juss. subsp. *villosa*	0.0557	10.7	0.214	1.45
L103	M. C. Li 103	*Patrinia monandra* C. B. Clarke	0.0543	8.0	0.159	1.64
L105	M. C. Li 105	*Patrinia monandra* C. B. Clarke	0.0517	11.5	0.229	1.46
L119	M. C. Li 119	*Patrinia scabiosifolia* Link	0.0531	20.1	0.402	1.44
L121	M. C. Li 121	*Patrinia scabiosifolia* Link	0.0544	10.7	0.213	1.28
L403	M. C. Li 403	*Patrinia villosa* (Thunb.) Juss. subsp. *villosa*	0.0540	28.7	0.574	1.81
L435	M. C. Li 435	*Patrinia monandra* C. B. Clarke	0.0539	31.4	0.629	1.28
L436	M. C. Li 436	*Patrinia monandra* C. B. Clarke	0.0575	12.3	0.247	1.51
L437	M. C. Li 437	*Patrinia scabiosifolia* Link	0.0518	6.8	0.136	1.66
L438	M. C. Li 438	*Patrinia scabiosifolia* Link	0.0608	8.4	0.168	1.46
L464	M. C. Li 464	*Patrinia villosa* (Thunb.) Juss. subsp. *villosa*	0.0509	16.1	0.322	1.53
L465	M. C. Li 465	*Patrinia villosa* (Thunb.) Juss. subsp. *villosa*	0.0302	27.1	0.541	1.39
L466	M. C. Li 466	*Patrinia heterophylla* Bunge	0.0280	10.9	0.217	1.47
L467	M. C. Li 467	*Patrinia heterophylla* Bunge	0.0610	9.2	0.185	1.57
L469	M. C. Li 469	*Patrinia heterophylla* Bunge	0.0538	29.5	0.590	1.58
L471	M. C. Li 471	*Patrinia heterophylla* Bunge	0.0561	35.5	0.710	2.33
L472	M. C. Li 472	*Patrinia villosa* (Thunb.) Juss. subsp. *villosa*	0.0562	40.6	0.813	1.68
L473	M. C. Li 473	*Patrinia villosa* (Thunb.) Juss. subsp. *villosa*	0.0531	20.6	0.413	1.55
L475	M. C. Li 475	*Patrinia monandra* C. B. Clarke	0.0176	24.4	0.489	1.62
L476	M. C. Li 476	*Patrinia heterophylla* Bunge	0.0561	15.0	0.301	1.92
L477	M. C. Li 477	*Patrinia villosa* (Thunb.) Juss. subsp. *villosa*	0.0590	26.0	0.520	1.29
L478	M. C. Li 478	*Patrinia villosa* (Thunb.) Juss. subsp. *villosa*	0.0521	28.7	0.574	1.81
L479	M. C. Li 479	*Patrinia monandra* C. B. Clarke	0.0555	28.6	0.572	1.67
L480	M. C. Li 480	*Patrinia monandra* C. B. Clarke	0.0535	32.2	0.645	1.76
L481	M. C. Li 481	*Patrinia scabiosifolia* Link	0.0579	35.8	0.715	1.87
L482	M. C. Li 482	*Patrinia scabiosifolia* Link	0.0556	8.8	0.176	1.95
L483	M. C. Li 483	*Patrinia scabra* Bunge	0.0542	19.5	0.389	1.72
L484	M. C. Li 484	*Patrinia villosa* (Thunb.) Juss. subsp. *punctifolia* H. J. Wang	0.0542	22.5	0.450	1.81
Z22076	X. L. Zhao 22076	*Patrinia scabiosifolia* Link	0.0523	24.5	0.490	1.56

Table S3 – NCBI GenBank accession number for the sequences generated from this work

Collector no.	Species	*atpB*	*petA*	*psaI-ycf4*	*rpl2-rpl23*	ITS2	*psbA-trnH*
M. C. Li 082	*Patrinia scabiosifolia* Link	PP280910	PP280930	PP280950	PP280970	PP277667	PP281007
M. C. Li 083	*Patrinia scabiosifolia* Link	OR712171	OR712188	OR712205	OR712222	PP277695	PP280998
M. C. Li 088	*Patrinia heterophylla* Bunge	PP280905	PP280925	PP280945	PP280965	PP277662	PP281002
M. C. Li 089	*Patrinia heterophylla* Bunge	OR712172	OR712189	OR712206	OR712223	PP277696	PP280999
M. C. Li 092	*Patrinia villosa* (Thunb.) Juss. subsp. *villosa*	PP280911	PP280931	PP280951	PP280971	PP277668	PP281008
M. C. Li 094	*Patrinia villosa* (Thunb.) Juss. subsp. *villosa*	PP280912	PP280932	PP280952	PP280972	PP277669	PP281009
M. C. Li 095	*Patrinia villosa* (Thunb.) Juss. subsp. *villosa*	PP280906	PP280926	PP280946	PP280966	PP277663	PP281003
M. C. Li 096	*Patrinia villosa* (Thunb.) Juss. subsp. *villosa*	PP280913	PP280933	PP280953	PP280973	PP277670	PP281010
M. C. Li 097	*Patrinia villosa* (Thunb.) Juss. subsp. *villosa*	PP280914	PP280934	PP280954	PP280974	PP277671	PP281011
M. C. Li 103	*Patrinia monandra* C. B. Clarke	OR712173	OR712190	OR712207	OR712224	PP277697	PP281000
M. C. Li 105	*Patrinia monandra* C. B. Clarke	PP280907	PP280927	PP280947	PP280967	PP277664	PP281004
M. C. Li 119	*Patrinia scabiosifolia* Link	PP280908	PP280928	PP280948	PP280968	PP277665	PP281005
M. C. Li 121	*Patrinia scabiosifolia* Link	PP280909	PP280929	PP280949	PP280969	PP277666	PP281006
M. C. Li 403	*Patrinia villosa* (Thunb.) Juss. subsp. *villosa*	OR712174	OR712191	OR712208	OR712225	PP277698	PP281001
M. C. Li 435	*Patrinia monandra* C. B. Clarke	PP280915	PP280935	PP280955	PP280975	PP277672	PP281012
M. C. Li 436	*Patrinia monandra* C. B. Clarke	PP280916	PP280936	PP280956	PP280976	PP277673	PP281013
M. C. Li 437	*Patrinia scabiosifolia* Link	PP280917	PP280937	PP280957	PP280977	PP277674	PP281014
M. C. Li 438	*Patrinia scabiosifolia* Link	PP280918	PP280938	PP280958	PP280978	PP277675	PP281015
M. C. Li 464	*Patrinia villosa* (Thunb.) Juss. subsp. *villosa*	PP280919	PP280939	PP280959	PP280979	PP277676	PP281016
M. C. Li 465	*Patrinia villosa* (Thunb.) Juss. subsp. *villosa*	PP280920	PP280940	PP280960	PP280980	PP277677	PP281017
M. C. Li 466	*Patrinia heterophylla* Bunge	PP280921	PP280941	PP280961	PP280981	PP277678	PP281018
M. C. Li 467	*Patrinia heterophylla* Bunge	PP280922	PP280942	PP280962	PP280982	PP277679	PP281019
M. C. Li 469	*Patrinia heterophylla* Bunge	OR712158	OR712175	OR712192	OR712209	PP277682	PP280985
M. C. Li 471	*Patrinia heterophylla* Bunge	OR712159	OR712176	OR712193	OR712210	PP277683	PP280986
M. C. Li 472	*Patrinia villosa* (Thunb.) Juss. subsp. *villosa*	OR712160	OR712177	OR712194	OR712211	PP277684	PP280987
M. C. Li 473	*Patrinia villosa* (Thunb.) Juss. subsp. *villosa*	OR712161	OR712178	OR712195	OR712212	PP277685	PP280988
M. C. Li 475	*Patrinia monandra* C. B. Clarke	OR712162	OR712179	OR712196	OR712213	PP277686	PP280989
M. C. Li 476	*Patrinia heterophylla* Bunge	OR712163	OR712180	OR712197	OR712214	PP277687	PP280990
M. C. Li 477	*Patrinia villosa* (Thunb.) Juss. subsp. *villosa*	OR712164	OR712181	OR712198	OR712215	PP277688	PP280991
M. C. Li 478	*Patrinia villosa* (Thunb.) Juss. subsp. *villosa*	OR712165	OR712182	OR712199	OR712216	PP277689	PP280992
M. C. Li 479	*Patrinia monandra* C. B. Clarke	OR712166	OR712183	OR712200	OR712217	PP277690	PP280993
M. C. Li 480	*Patrinia monandra* C. B. Clarke	OR712167	OR712184	OR712201	OR712218	PP277691	PP280994
M. C. Li 481	*Patrinia scabiosifolia* Link	OR712168	OR712185	OR712202	OR712219	PP277692	PP280995
M. C. Li 482	*Patrinia scabiosifolia* Link	OR712169	OR712186	OR712203	OR712220	PP277693	PP280996
M. C. Li 483	*Patrinia scabra* Bunge	PP280923	PP280943	PP280963	PP280983	PP277680	PP281020
M. C. Li 484	*Patrinia villosa* (Thunb.) Juss. subsp. *punctifolia* H. J. Wang	PP280924	PP280944	PP280964	PP280984	PP277681	PP281021
X. L. Zhao 22076	*Patrinia scabiosifolia* Link	OR712170	OR712187	OR712204	OR712221	PP277694	PP280997
